# Expression of transgenes enriched in rare codons is enhanced by the MAPK pathway

**DOI:** 10.1038/s41598-020-78453-5

**Published:** 2020-12-17

**Authors:** Jackson Peterson, Siqi Li, Erin Kaltenbrun, Ozgun Erdogan, Christopher M. Counter

**Affiliations:** 1 Department of Pharmacology and Cancer Biology, Duke University Medical Center, Durham, NC 27710 UK; 2 Department of Radiation Oncology, Duke University Medical Center, Durham, NC 27710 UK

**Keywords:** Molecular biology, Translation

## Abstract

The ability to translate three nucleotide sequences, or codons, into amino acids to form proteins is conserved across all organisms. All but two amino acids have multiple codons, and the frequency that such synonymous codons occur in genomes ranges from rare to common. Transcripts enriched in rare codons are typically associated with poor translation, but in certain settings can be robustly expressed, suggestive of codon-dependent regulation. Given this, we screened a gain-of-function library for human genes that increase the expression of a GFP^rare^ reporter encoded by rare codons. This screen identified multiple components of the mitogen activated protein kinase (MAPK) pathway enhancing GFP^rare^ expression. This effect was reversed with inhibitors of this pathway and confirmed to be both codon-dependent and occur with ectopic transcripts naturally coded with rare codons. Finally, this effect was associated, at least in part, with enhanced translation. We thus identify a potential regulatory module that takes advantage of the redundancy in the genetic code to modulate protein expression.

## Introduction

A universal feature in all kingdoms is genetic redundancy, namely 61 nucleotide triplets, or codons, code for only 20 canonical amino acids^1^. Attempts to reduce the number of codons in a genome can reduce organismal fitness^2^, arguing that this genetic redundancy is essential for life. Codons encoding the same amino acid, or synonymous codons, occur at different frequencies within the coding regions of the genome, ranging from rare to common^3^. What is considered rare or common is conserved across closely related species, but divergent across larger evolutionary distances^3,4^. In mammals, codons ending in either A or T are typically rare while those ending in either C or G are common^5^. A bias towards rare codons is associated with poor translation, which is thought to result from differential expression of the cognate tRNA species^4^. Consequently, rare codons may cause the ribosome to pause while waiting for entry of the underrepresented correct tRNA and/or while rejecting the entry of near-cognate tRNAs^6–8^. Such pausing can slow translation elongation^4,9^, which in turn can inhibit translational initiation^10^, promote mRNA degradation^11^, and even affect protein folding^12,13^. Evolutionarily conserved clusters of rare codons have also been associated with boundaries of folding domains^14,15^ and co-translational folding intermediates^16^, arguing that specifically placed rare codons can also fine-tuned translation kinetics to facilitate efficient protein folding.

As a bias towards rare codons is often associated with poor protein production, codon usage is often manipulated to optimize protein expression in heterologous systems, for example expressing a recombinant human protein in bacteria^17^. Beyond these artificial settings, there are examples whereby modifying codon usage at the endogenous locus imparts predictable effects on protein expression in *E. coli*^18,19^, *Drosophila*^20^*,* and mice^21^, to name a few. Specifically, changing naturally occurring rare codons to their common counterparts increases protein expression while converting common codons to rare has the opposite effect. These trends are confirmed by in vitro translation experiments^18,22^ and by ribosomal profiling approaches to measure ribosomal occupancy at a transcriptome-wide level^6–8^. Codon usage is therefore an intrinsic feature of gene expression.

In an apparent contradiction to the above, there are settings whereby rare codon-enriched transcripts are robustly expressed. Focusing on mammals, certain genes with non-optimal codons are preferentially expressed during G2/M phase of cell cycle in human cells^23^. Some human cancer cell lines express high levels of KRAS protein^24^, even though the mRNA is enriched in rare codons^25^. Certain viral genes with a bias towards the rare codons of their mammalian host are highly expressed in the host cells^26–29^. In fact, vertebrate interferon responses appear to have evolved mechanisms to counter the biosynthetic needs of codon-biased viruses^30,31^, and in response, viral proteins appear to evolve codon usage that matches the tRNA expression profiles of interferon-treated cells^32^.

The above findings suggest mechanisms to overcome poor translation dictated by rare codons, potentially to regulate protein levels based on codon usage. There is evidence to suggest that changing the expression or charging of tRNAs can modulate the expression of genes in a codon-dependent fashion. Relative tRNA levels vary across tissues^33^, and tRNA levels correlate with the codon usage of highly expressed genes during proliferation and differentiation processes^34^. Indeed, somatic cell reprogramming involves a C-MYC driven tRNA program^35^. Conversely, under conditions of amino acid deprivation, normally under-represented tRNAs remain charged and enhance the translation of components of the ubiquitin proteasomal system^36^.

Beyond tRNA, proteins may also affect expression of mammalian genes depending on codon usage. Perhaps the best example is the anti-viral host protein SLFN11, which cleaves type II tRNAs and reduces translation of transcripts with the rare leucine TTA codon, like that found in HIV^30^ or the DNA damage sensor ATR kinase^37^. Given the effect of rare codon bias on gene function, the increasing number of examples of rare codon-enriched transcripts highly expressed, and at least one tangible example of a mammalian protein regulating translation in a rare codon-dependent manner, we sought to identify pathways that affect rare codon-dependent expression. To this end, we hypothesized that a fluorescent protein coded with rare codons could be used to detect rare codon-dependent expression. Further, as certain human cancer cell lines have been documented to express high levels of rare codon-enriched transcripts^24^, it stands to reason that pathways altered in this disease could serve as a starting point to screen for such factors. To therefore identify modifiers of rare codon-dependent expression in mammals we screened a cDNA library encoding common cancer-related proteins for their ability to preferentially increase the expression of a fluorescent reporter enriched in rare codons in human cells.

## Results

### A reporter system for rare codon-dependent expression

**Figure 1:**
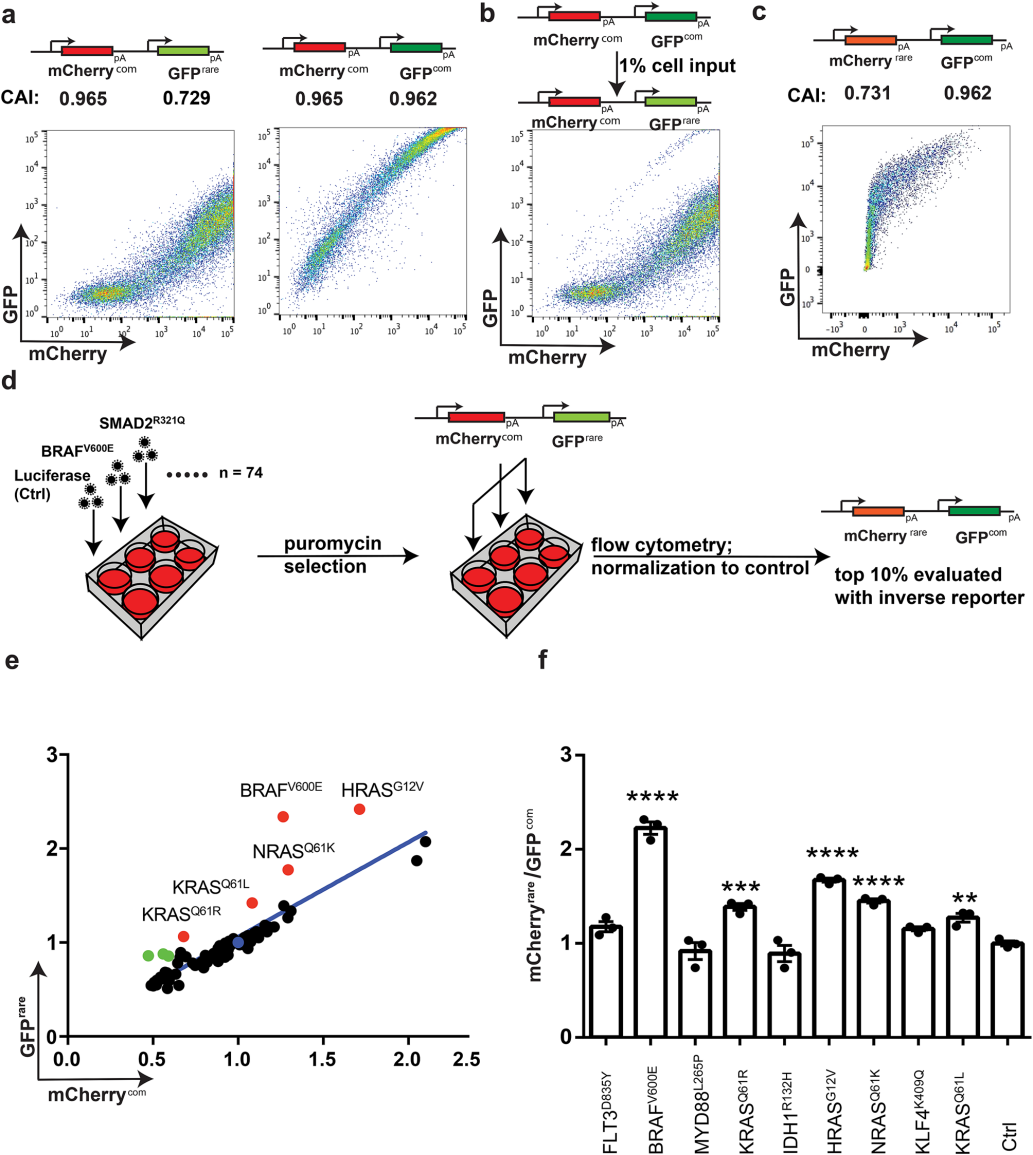
An unbiased screen for enhancers of rare codon-enriched expression. (**a**–**c**) Design of the reporters and their CAI (top) as well as the degree of GFP versus mCherry fluorescence, as assessed by FACS, of 293T cells transfected with the indicated reporters (bottom). One of three or four biological replicates. (**d**) Schematic of CTK codon-dependent fluorophore screen. (**e**) Scatter plot of MFI of GFP^rare^ versus mCherry^com^ normalized to the fluorescence of parallel luciferase-control cells for each CTK screen. Blue dot: normalized control. Green dots: top 10%. Red dots: top 10% validated (labeled) in secondary screen with the mCherry^rare^: GFP^com^ reporter. Line: Regression line of the data. (**f**) Mean ± SEM of the ratio of mCherry^rare^ to GFP^com^ fluorescence normalized to the fluorescence of parallel luciferase control cells for the indicated CTK-infected 293T cells strains determined from three technical replicates. *P* values were calculated from comparison against Ctrl. Ctrl: cells expressing luciferase control. One-way ANOVA with Sidak’s multiple comparisons test: **P < 0.01; ***P < 0.001 and ****P < 0.0001.

To screen for pathways modifying expression in a rare codon-dependent fashion in mammals, we developed a fluorescent reporter system amenable to high-throughput analysis that was capable of distinguishing protein levels based on codon usage. An expression vector containing a Green Fluorescent Protein (GFP) cDNA with a codon bias towards rare mammalian codons (GFP^rare^) was generated (Supplementary Figs. 1a, 2a) based on the original *A. victorius* sequence with substitutions that improved fluorescence^38^, somewhat akin to the approach used to screen for amino acid overproducers in bacteria^39^. For comparison, the converse was also developed (Supplementary Figs. 1b, 2a), namely the same vector encoding EGFP cDNA optimized for common mammalian codons (GFP^com^), which is well established to be expressed at high levels in human cells^38,40^. The human 293T cell line, chosen for its ease as a high-throughput cellular screening platform^41^, was transfected with either of these two vectors. The resultant two populations were then subjected to Fluorescence-Activated Cell Sorting (FACS) analysis to separate cells based on the level of their GFP fluorescence. As expected, GFP^rare^ exhibited approximately 100-fold lower Mean Fluorescent Intensity (MFI) when compared to GFP^com^. Despite this obvious difference in expression, GFP^rare^-expressing cells were not fully resolvable from their GFP^com^ counterparts (Supplementary Fig. 2b). To overcome this limitation, mCherry^com^ cDNA encoding the fluorescent protein mCherry enriched in common codons (Supplementary Figs. 1c, 2c) was inserted into these vectors to allow for normalization of expression levels. 293T cells transfected with the mCherry^com^:GFP^rare^ or mCherry^com^:GFP^com^ reporter constructs were again subjected to FACS analysis, revealing a linear relationship between mCherry^com^ and GFP^rare^ or GFP^com^ fluorescence (Fig. 1a and Supplementary Fig. 2d,e). To validate that this reporter system effectively separates GFP^rare^ and GFP^com^ cells, we mixed the two populations and showed that mCherry^com^:GFP^com^ cells were captured at high efficiency when spiked in at as little as 1% of the mCherry^com^:GFP^rare^ population (Fig. 1b). Finally, to confirm that this effect was dependent upon codon usage and not protein sequence, the experiment was repeated using a vector encoding mCherry^rare^ coded with rare codons and GFP^com^ (Supplementary Figs. 1d, 2c). Cells transfected with this reporter exhibited the reverse FACS pattern (Fig. 1c). Thus, the combination of this reporter system with a FACS-based platform enables efficient separation of common and rare codon-enriched fluorescent proteins in a manner independent of protein sequence.

### A gain-of-function screen for modifiers of rare codon-dependent expression

We next sought to identify proteins affecting the ratio of GFP^rare^ to mCherry^com^ fluorescence. Given that a subset of cancer cells exhibit elevated expression of tested rare codon-enriched transcripts^24^, it seemed plausible that one or more of the pathogenic pathways of cancer cells may underlie this effect. We thus turned to a gain-of-function Cancer Toolkit (CTK) lentiviral library in which the major proliferative and other pro-cancer pathways are represented by multiple mutant or over-expressed proteins^42,43^. 293T cells were stably infected with each of 74 different CTK lentiviruses encoding a different cancer protein (Supplementary Table 1), or as a control, a lentivirus encoding luciferase. These cell lines were then transfected with the mCherry^com^:GFP^rare^ reporter and subjected to FACS analysis (Fig. 1d). The MFI of GFP^rare^ and mCherry^com^ for each cell line was then normalized to the luciferase-infected cells transfected with the same vector, and reported as a ratio of GFP^rare^ to mCherry^com^ normalized MFI, termed relative GFP^rare^ for simplicity. This analysis identified nine candidate CTK genes exhibiting the highest (top 10%) increase in relative GFP^rare^ (Fig. 1e and Supplementary Table 1). To rule out effects due to protein sequence, we retested those CTK cells lines that exhibited the highest (top 10%) increase in relative GFP^rare^ expression with the mCherry^rare^:GFP^com^ reporter and set an arbitrary cut-off to be an increase in relative mCherry^rare^ expression by at least 20%. This reduced the number of candidates to five cell lines that exhibited an increase in both relative GFP^rare^ and relative mCherry^rare^ expression (Fig. 1f). We define these as candidate enhancers of rare codon-dependent expression.

### The MAPK pathway enhances GFP^rare^ expression

**Figure 2:**
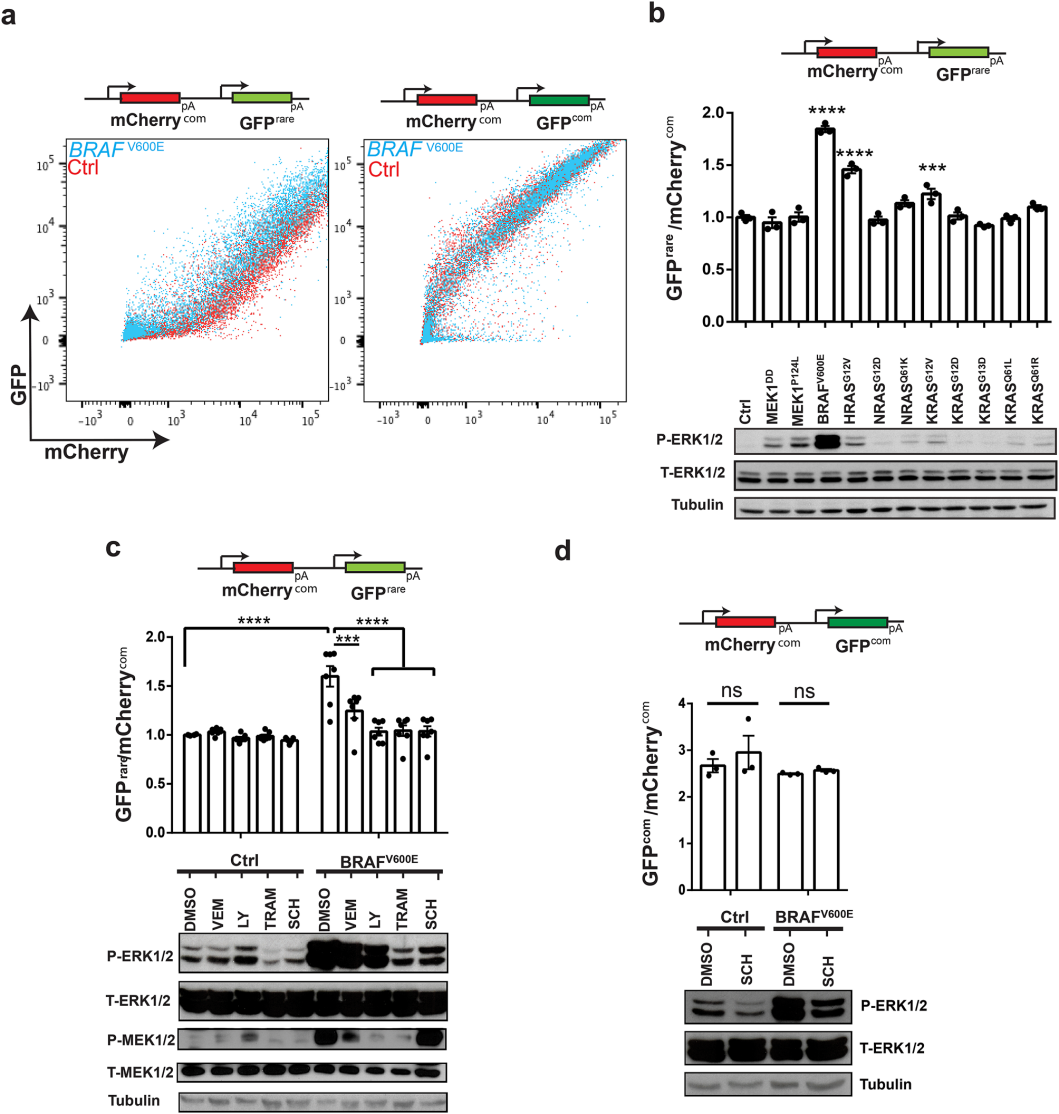
The MAPK pathway enhances GFPrare expression. (**a**) Reporter used (top) and degree of GFP versus mCherry fluorescence, as assessed by FACS, of 293T cells stably expressing either control (Ctrl) luciferase (red dots) or BRAF^V600E^ (blue dots) when transfected with the indicated reporters (bottom). One of three biological replicates. (**b**–**d**) Reporters used (top), mean ± SEM of the ratio of (**b**,**c**) GFP^rare^ to mCherry^com^ or (**d**) GFP^com^ to mCherry^com^ fluorescence normalized to the fluorescence of parallel luciferase control (Ctrl) cells for three technical replicates (middle), and immunoblot analysis of phosphorylated (P) and total (T) ERK1/2 and when tested MEK1/2, with tubulin serving as a loading control (bottom), for 293T cells stably expressing the luciferase control (Ctrl) and (**b**) the indicated oncogenes or (**c**,**d**) BRAF^V600E^ when transfected with the indicated reporter and, when denoted, after treatment with MAPK inhibitors: vemurafenib (vem), LY3009120 (LY), trametinib (TRAM) or SCH772984 (SCH). One of two or three biological replicates. *P* values in (**b**) were calculated from comparison against luciferase control. One-way (**b**) or two-way ANOVA (**c**,**d**) with Sidak’s multiple comparisons test: ns: *P* ≥ 0.5, ***P < 0.001 and ****P < 0.0001.

All five of the candidate genes identified to be enhancers of rare codon-dependent expression are oncogenes that activate the mitogen activated protein kinase (MAPK) pathway. This pathway is comprised of the initiating A, B, or CRAF kinases that phosphorylate and activate the MEK1/2 kinase, which in turn phosphorylate and activate terminal ERK1/2 kinases^44^. The top candidate was BRAF^V600E^ (Figs. 1e,f, 2a and Supplementary Table 1), a constitutively active and oncogenic version of this kinase^45^. This was followed by oncogenic mutants of all three RAS isoforms, namely HRAS^G12V^, NRAS^Q61K^, KRAS^Q61R^, and KRAS^Q61L^, although admittedly not every one of the oncogenic RAS mutants in the CTK library were identified in this screen (Figs. 1e,f, 2a and Supplementary Table 1). As RAS oncoproteins are well known to directly bind to and activate RAF kinases^46^, this further suggest that the MAPK pathway enhances expression in a rare codon-dependent manner. To explore this possibility further, 293T cells were stably infected with lentiviruses expressing luciferase as a control, the identified five transgenes, other oncogenic versions of RAS included in the CTK library, or activated versions of MEK1 kinase. These 11 stable cells were then transiently transfected with the mCherry^com^:GFP^rare^ reporter. Immunoblot analysis revealed that these oncogenes variably increased the phosphorylation of T202 and Y204 on ERK1/2, termed P-ERK1/2 (Fig. 2b), indicative of an activated MAPK pathway^47^. Of all the tested proteins, BRAF^V600E^ and HRAS^G12V^ exhibited the largest increase in P-ERK1/2 and relative GFP^rare^ (Fig. 2b), suggesting that particularly potent activation of the MAPK pathway has the greatest effect on relative GFP^rare^ expression. Of note, MEK1^P124L^ and MEK^DD^ efficiently increased P-ERK1/2 levels but did not alter relative GFP^rare^ (Fig. 2b). With these two noted exceptions, these findings are consistent with the MAPK pathway enhancing expression in a rare codon-dependent fashion.

### Inhibiting the MAPK pathway suppresses GFP^rare^ expression

To interrogate the effect of MAPK signaling on relative GFP^rare^ levels, 293T cells stably infected with a lentivirus encoding BRAF^V600E^ or control luciferase were transfected with the mCherry^com^:GFP^rare^ reporter and treated with vehicle (DMSO) or the small molecule inhibitors LY3009120, which targets the RAF family of kinases^48^, vemurafenib, which targets the oncogenic BRAF^V600E^ version of BRAF^49^, trametinib, which targets the MEK1/2 kinases^50^, or SCH772984, which targets the ERK1/2 kinases^51^. Following 24 h of treatment with these inhibitors, the level of P-ERK1/2, phosphorylated MEK1/2 (termed P-MEK1/2), and relative GFP^rare^ were determined by immunoblot and FACS analysis, respectively. As expected, the luciferase-infected cells had both low MAPK signaling, as evidenced by minimal P-ERK1/2 and P-MEK1/2 levels, and low relative GFP^rare^ expression, while the BRAF^V600E^-infected cells had high levels of both MAPK signaling and relative GFP^rare^. Treating the latter cells with the described MAPK inhibitors variably reduced P-MEK1/2, P-ERK1/2, and relative GFP^rare^ levels (Fig. 2c). The only exception was SCH772984, which still reduced P-ERK1/2 and relative GFP^rare^ levels (Fig. 2c), but led to elevated P-MEK1/2, consistent with the known effect of this drug stimulating a feed-back mechanism activating RAF kinases^52^. To independently validate these findings, the entire experiment was repeated, except the MAPK pathway was activated with two different oncogenic RAS isoforms (HRAS^G12V^ or KRAS^Q61R^) in the absence and presence of SCH772984, with the same outcome, namely a reduction in both MAPK signaling and relative GFP^rare^ levels (Supplementary Fig. 3a). To address whether this effect depends upon rare codons, the experiment was repeated with the mCherry^com^:GFP^com^ reporter in the absence and presence of the ERK1/2 inhibitor, which found that SCH772984-mediated inhibition of the MAPK pathway did in fact not alter relative GFP^com^ levels (Fig. 2d). Thus, inhibiting the MAPK pathway suppresses expression in a rare codon-dependent fashion.

### The MAPK pathway enhances GFP expression dependent upon the degree of rare codon usage

**Figure 3:**
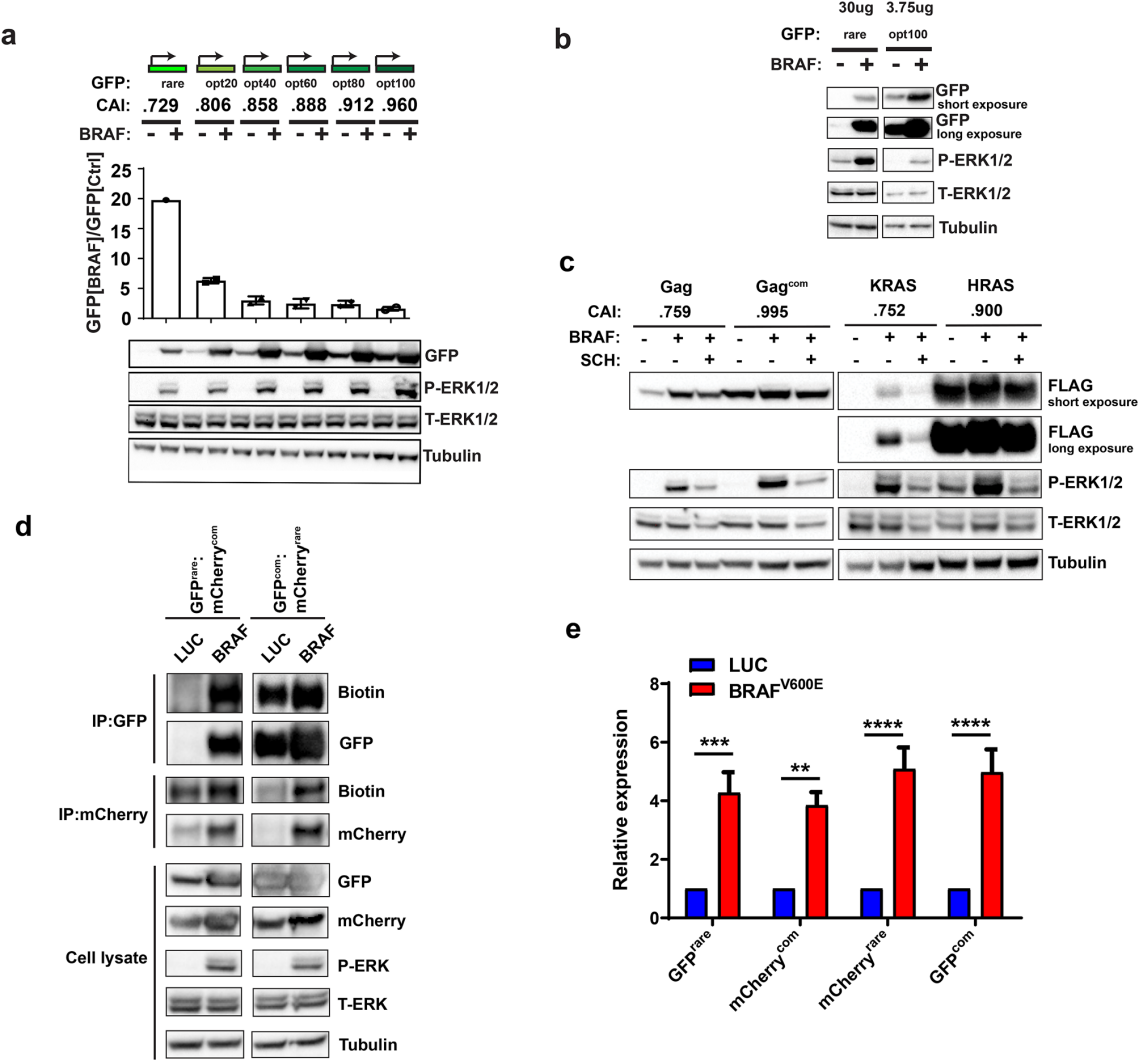
The MAPK pathway enhances expression dependent on the degree of rare codon usage. (**a**) Reporters used and their CAI (top), mean ± SEM of the ratio of GFP fluorescence of 293 cells stably expressing BRAF^V600E^ versus the luciferase control (Ctrl) from two independent experiments (middle), and a representative immunoblot analysis GFP, P-ERK1/2, T-ERK1/2, and tubulin (bottom) from the same cells (n = 2). (**b**) Representative immunoblot analysis of GFP, P-ERK1/2, T-ERK1/2, and tubulin of lysates from 293 cells co-transfected with vectors encoding either control luciferase (−) or BRAF^V600^E (+) and the GFP reporter with 0% or 100% common codons. Protein amount loaded is indicated on top. One of two biological replicates. Left and right blots (separated by white space) are from different gels in the same experiment. (**c**) Representative immunoblot analysis of FLAG-tagged Gag, Gag^com^, KRAS, HRAS, P-ERK1/2, T-ERK1/2, and tubulin from 293 cells co-transfected with a vector encoding either luciferase control (−) or BRAF^V600E^ (+) and the indicated FLAG-tagged proteins in the absence (−) or presence (+) of the ERK1/2 inhibitor SCH772984 (SCH). One of two biological replicates. Left and right blots (separated by white space) are from different gels in the same experiment. (**d**) Immunoblot analysis of AHA-biotin labeled mCherry or GFP in 293T cells transiently expressing mCherry^com^ and GFP^rare^ or mCherry^rare^ and GFP^com^ in the presence of BRAF^V600E^ (BRAF) or control luciferase (Luc). One of two biological replicates. Left and right blots (separated by white space) are from different parts of the same gel at different exposures, except for P-ERK1/2, T-ERK1/2 and Tubulin, which are different parts of the same gel at the same exposure. (**e**) Mean ± SEM of the fold change of *mCherry* or *GFP* mRNA, as assessed by RT-PCR analysis of four independent experiments, of 293T cells transiently expressing mCherry^com^ and GFP^rare^ or mCherry^rare^ and GFP^com^ in the presence BRAF^V600E^ versus luciferase control (Luc). Two-way ANOVA with Sidak’s multiple comparisons test: **P < 0.01; ***P < 0.001 and ****P < 0.0001.

To explore the relationship of the MAPK pathway on rare codon-dependent expression, a series of six expression vectors were created in which the codon usage of GFP^rare^ was progressively altered towards 100% optimality by converting rare codons to common for specific amino acids (Supplementary Fig. 1e–i). 293 cells were co-transfected with each of these vectors and a vector encoding either BRAF^V600E^ to activate the MAPK pathway or luciferase as a control. Following this, the GFP levels of the transfected cells were assessed by immunoblot. The greatest increase in GFP expression induced by BRAF^V600E^ was observed with GFP containing 0% optimized codons (GFP^rare^), and to a lesser extent, 20% optimized codons (GFP^opt20^) (Fig. 3a). For a better comparison, the experiment was repeated with GFP^rare^, GFP^opt20^, and GFP^opt100^ at different protein concentrations and exposures, demonstrating that GFP^rare^ and GFP^opt20^ expression (e.g*.* as observed with high protein concentration, long exposure) were clearly increased more than GFP^opt100^ (e.g. as observed with low protein concentration, short exposure) in cells transfected with the vector encoding BRAF^V600E^ (Fig. 3b and Supplementary Fig. 3b). These findings suggest that the degree of rare codon usage influences the amount that the MAPK pathway can increase protein expression.

### The MAPK pathway enhances expression of mRNA with a natural rare codon bias

To assess the effect on native transcripts with a bias towards rare codons we turned to two well-documented cases whereby a rare codon bias has been shown to impede protein expression in human cells, the *Gag* gene of HIV^53^ and the *KRAS* gene of humans^25^. The *Gag* gene is enriched in rare codons of the host human cells and changing these to their common counterparts (*Gag*^*com*^) increases protein expression in tested human cells^26^. Similarly, *KRAS* is enriched in rare codons and poorly expressed in a number of tested human cells while the highly related isoform *HRAS* is enriched in common codons and robustly expressed^25^. 293 cells were co-transfected with reporters encoding N-terminally FLAG-tagged Gag, Gag^com^, KRAS, or HRAS and an expression vector encoding either BRAF^V600E^ or luciferase as a control (Supplementary Figs. 1j–m, 3c,d). Immunoblot analysis revealed that BRAF^V600E^ appropriately activated the MAPK pathway, as evidenced by increased P-ERK1/2, but also increased Gag and KRAS protein expression. Further, inhibiting BRAF^V600E^ activation of the MAPK pathway with the ERK1/2 inhibitor SCH772984, as assessed by a reduction in P-ERK1/2 levels, reduced the increase in Gag and KRAS protein expression induced by BRAF^V600E^. We note there was also an increase in Gag^com^ and HRAS protein levels in cells transfected with the BRAF^V600E^ expression vector, although to a lesser degree, and the effect was less sensitive to MAPK inhibition (Fig. 3c and Supplementary Fig. 4a). These findings suggest the MAPK pathway generally enhances protein expression, but this is more pronounced with transcripts naturally enriched in rare codons.

### The MAPK pathway increases GFP^rare^ translation

While codon usage can affect all steps of protein synthesis, changes in codon usage are well established to alter translation initiation and elongation^4,9,10^, and thus translation represents a good starting point for mechanistic studies. To this end we measured the amount of newly synthesized rare versus common codon-enriched reporter proteins upon stimulation of the MAPK pathway. 293T cells were co-transfected with expression vectors encoding mCherry^com^:GFP^rare^, mCherry^rare^:GFP^com^, or as a control no transgene, and either BRAF^V600E^ to activate the MAPK pathway or as a control luciferase. Cells were then cultured in methionine-free media that was then supplemented with l-Azidohomoalanine (AHA) for 120 min to label newly synthesized proteins, or as a control, methionine. GFP and mCherry proteins were immunoprecipitated with an anti-GFP or anti-mCherry antibody and treated with DBCO-biotin to conjugate biotin to AHA-labeled protein. Immunoprecipitates were immunoblotted with an anti-GFP or anti-mCherry antibody to detect total protein or an anti-biotin antibody to detect newly synthesized protein. Compared to luciferase control cells, BRAF^V600E^-transduced cells exhibited elevated P-ERK1/2, indicative of activation of the MAPK pathway, and elevated levels of all four fluorescent reporter proteins (Fig. 3d and Supplementary Fig. 4b). With regards to new synthesized protein, BRAF^V600E^-transduced cells exhibited a general increase in AHA-labelled proteins compared to control luciferase-transduced cells (Supplementary Fig. 4c), and consistent with these findings, AHA labeling of all four fluorescent reporter proteins was elevated in the BRAF^V600E^-transduced cells (Fig. 3d and Supplementary Fig. 4b). AHA labeling was confirmed to reflect newly synthesized protein, as immunoprecipitated GFP and mCherry were not detected with the anti-biotin antibody in cells treated with methionine (Supplementary Fig. 4d). The increase of AHA-labeled GFP^rare^, and to a lesser extent mCherry^rare^ protein, from luciferase control to BRAF^V600E^-transduced cells was more significant than AHA-labeled GFP^com^ and mCherry^com^ protein, respectively (Fig. 3d and Supplementary Fig. 4b). Note that AHA-labeled GFP^rare^/mCherry^rare^ and immunoprecipitated GFP^rare^/mCherry^rare^ in luciferase control cells are detectable when the blot is sufficiently overexposed (data not shown). To assess whether this increase was also a product of elevated transcription, the mRNA of the four fluorescent reporters was determined by qRT-PCR in the absence and presence of BRAF^V600E^. While there was variation from experiment to experiment, on average, *GFP*^*rare*^, *GFP*^*com*^, *mCherry*^*rare*^, and *mCherry*^*com*^ mRNA levels all increased similarly in cells transduced with the BRAF^V600E^ expression vector (Fig. 3e). In conclusion, while activation of the MAPK pathway led to a general increase in mRNA and protein synthesis, there was a reproducible increase in the synthesis of rare-codon reporters compared to their common counterparts. These findings point towards translation underlying at least part of the preferential increase in rare-codon enriched expression induced by the MAPK pathway.

## Discussion

As noted above, a variety of settings have been documented whereby a rare codon-enriched gene is, contrary to its codon usage, robustly expressed in mammals. As there was little insight into the underlying mechanism of this effect in mammals, we screened for pathways capable of increasing the expression of the rare codon-enriched reporter GFP^rare^. Here we demonstrate that an unbiased screen identified the MAPK pathway as an enhancer of rare codon-dependent expression. Gain- and loss-of-function validation experiments further confirm that stimulating the MAPK pathway increases GFP^rare^ expression in a codon-dependent manner, and that this effect is transferable to other rare codon-enriched transcripts. One exception was the MEK mutants MEK1^P124L^ and MEK^DD^, which increase P-ERK1/2 without preferentially increasing GFP^rare^. Perhaps related, one or both of these mutants have previously been reported to be less oncogenic^54^ and/or reduced in their ability to induce global protein synthesis^55^ compared to BRAF^V600E^. However, we cannot rule out the involvement of other proteins aside from ERK1/2 kinases mediating the higher expression of GFP^rare^ protein. This screen was agnostic to how relative GFP^rare^ levels were altered, and changes in codon usage have been documented to affect transcription^56,57^, mRNA stability^11,58,59^, translation initiation and elongation^4,9,10^, and even protein folding and stability^12,13^. While stimulating the MAPK pathway most certainly increases the amount of the mRNA encoded by the various fluorescent reporters, it did so apparently independent of codon usage. Conversely, we observe a larger increase in AHA-labelled GFP^rare^, and to a lesser extent mCherry^rare^ proteins in BRAF^V600E^-transfected cells compared to their common codon-enriched counterparts. Collectively, these studies implicate translation, although we did not rule out an effect on protein folding.

Mechanistically, we suggest that activating the MAPK pathway results in a general increase in translation, but rare codon-enriched transcripts have a larger dynamic range. Indeed, ERK1/2 are known to phosphorylate proteins that promote translation. Namely, MNK1/2 kinases^60^, which phosphorylate eIF4E^61^, and RSK kinases^62^, which phosphorylate rpS6^63^ and eIF4B^64^. ERK1/2 also phosphorylate the BRF1 subunit of transcription factor TFIIIB, which increases tRNA synthesis^65^. More indirectly, ERK1/2 signaling enhances c-Myc protein stability^66^, which stimulates the transcription of a number of genes encoding proteins necessary for ribosome biogenesis^67,68^. ERK1/2 also activate mTORC1, which promotes protein synthesis through phosphorylation of effectors including 4E-BP1 and S6K1^69^, by phosphorylating TSC2^70^ or Raptor^71^. Any one or more of these effects could be at play, although we cannot discount a preferential increase specifically in the translation of rare codon-enriched mRNA. Regardless, in either scenario codon bias is the deciding factor.

One logical extension of our findings is that the MAPK pathway may modulate the composition of the proteome based on codon usage, and even more speculative, that codon bias is a regulated feature hardwired into the very sequence of the genome. Empirically we found that manipulating codon usage has the greatest effect on highly represented transcripts^25^. As such, stimulating the MAPK pathway may not necessarily globally enhance expression of rare codon-enriched transcripts, but instead may be restricted to highly expressed genes, for example like those of certain viruses. Indeed, the MAPK pathway enhances expression of *Gag* in a manner sensitive to the rare codon bias of this gene, and the host protein SLFN11 has previously been shown to affect the expression of *Gag* in a codon-dependent manner^30^. We also find that the increase in expression of rare codon-enriched reporters was most pronounced with a combination of potent activation of the MAPK pathway and reporters with the rarest codon bias, which again may influence the type of transcripts and conditions leading to elevated expression. Nevertheless, the very fact that the MAPK pathway increases expression of multiple tested ectopic rare codon-enriched transcripts argues that at least in these settings, this pathway enhances rare codon-dependent expression, a novel observation. In conclusion, we document a rare codon-dependent increase in translation by the MAPK pathway in mammalian cells, opening the door to exploring the codon-dependent relationship between pro-proliferative signaling and protein translation.

## Methods

### Mammalian cell lines

293 and 293T cells were grown at 37 °C in 5% CO_2_ and cultured in DMEM supplemented with 10% FBS and 1% penicillin/streptomycin. All cell lines were purchased from American Type Culture Collection or Duke University Cell Culture Facility.

### Plasmids

pcDNA3.1 + GFP^rare^ was created by cloning *GFP*^*rare*^ cDNA that was synthesized (Thermo Fisher Scientific GeneArt) based on the sequence of *Aequoria Victoria*, with an EGFP amino acid substitution (S_65_T) to enhance fluorescence^38^. The resulting vector was validated through sequencing. pcDNA3.1 + GFP^com^ was created by PCR cloning *pEGFPc2* cDNA (Clontech) into pcDNA3.1+. The resulting vector was validated through sequencing. pcDNA3.1 + mCherry^rare^ was created by cloning *mCherry*^*rare*^ cDNA that was synthesized (Thermo Fisher Scientific GeneArt) based on the sequence of an *mCherry* cDNA (Addgene vector # 19327) codon-optimized for expression in *C. elegans*^72^, which contains low codon optimality in mammalian cells. The resulting vector was validated through sequencing. pcDNA3.1 + mCherry^com^ was created was created by PCR cloning *mCherry2-C1* cDNA (Addgene vector # 54563, Michael Davidson, unpublished) into the HindIII and BamHI sites of pcDNA3.1+. The resulting vector was validated through sequencing. pcDNA3.1 + mCherry^com^:GFP^rare^ was created by PCR cloning the expression cassette pcDNA3.1 + mCherry^com^ (including the CMV promoter, *mCherry*^*com*^ cDNA, and polyA region) into the BglII restriction site of pcDNA3.1 + GFP^rare^ and sequenced to validate proper content and orientation of the insert. pcDNA3.1 + mCherry^rare^:GFP^com^ was created by PCR cloning the expression cassette pcDNA3.1 + mCherry^rare^ (including the CMV promoter, *mCherry*^*rare*^ cDNA, and polyA region) into the BglII restriction site of pcDNA3.1 + GFP^com^ and sequenced to validate proper content and orientation of the insert. pcDNA3.1 + mCherry^com^:GFP^com^ was created by PCR cloning the expression cassette pcDNA3.1 + mCherry^com^ (including the CMV promoter, *mCherry*^*com*^ cDNA, and polyA region) into the BglII restriction site of pcDNA3.1 + GFP^com^ and sequenced to validate proper content and orientation of the insert. pcDNA3.1 + GAG was created by cloning *GAG* cDNA that was synthesized (Thermo Fisher Scientific GeneArt) based on the sequence of the HIV-1 HXB2 Gag-EGFP expression vector^73^ (NIH AIDS Reagent Program vector # 11468) with N-terminal FLAG tag and inserted into the pcDNA3.1 + expression vector in the BamHI and EcoRI restriction sites. The resulting vector was validated through sequencing. pcDNA3.1 + GAG^com^ was created by cloning *GAG*^*com*^ cDNA that was synthesized (Thermo Fisher Scientific GeneArt) based on p96ZM651.8 Gag sequence^38^ (NIH AIDS Reagent Program vector # 8675) with codon-optimized amino acid substitutions to match the exact amino acid sequence of *Gag*^*rare*^ with N-terminal FLAG tag and inserted into the pcDNA3.1+ expression vector in the BamHI and EcoRI restriction sites. The resulting vector was validated through sequencing. pcDNA3.1 + KRAS was PCR cloned from previously described pBabePuro-KRAS construct^25^. pcDNA3.1 + HRAS was PCR cloned from previously described pBabePuro-HRAS construct^25^. pLX303BRAF^V600E^ and pLX303Luciferase were cloned from pCW107^42^ to pLX303^74^ (Addgene vector # 25897) using Gateway recombination methods (Thermo Fisher Scientific). pCW107 vectors were mixed with the BP-Clonase reaction and the pDONR223^74^ entry vector (Addgene vector # 25894) and sequence verified to generate pDONR223 vectors. Next, a LR-Clonase reaction with pDONR223 entry vector and pLX303 destination vector was performed and vectors were validated through sequencing. pcDNA3.1 + GFP^opt20^, pcDNA3.1 + GFP^opt40^, pcDNA3.1 + GFP^opt60^, pcDNA3.1 + GFP^opt80^, and pcDNA3.1 + GFP^opt100^ were cloned using synthesized templates (Thermo Fisher Scientific GeneArt) encoding GFP sequences with various codon usage. The codons in native GFP were progressively changed to synonymous codons used in EGFP at an even pace (~ 30 codons a time, each time particular amino acids were optimized, LG + VK + TDE + ISP + FAQHNYR). The coding sequences for GFP were amplified by PCR using Q5 High-Fidelity DNA Polymerase (NEB), subcloned into the pcDNA3.1+ vector and sequencing validated. Codon adaptation index (CAI) of all cDNAs was calculated with the E-CAI tool^75^ according to a sliding, 3 codon, scale to demonstrate codon optimality differences along the length of the indicated transcripts.

### Cancer Toolkit v2 (CTKv2) library

Individual cDNAs (Supplementary Table 1) from an expanded version 2 (V2) of the Cancer Toolkit library^42^ were cloned with gateway technology into pCW107 lentiviral expression vector.

### Lentivirus generation and infection

Lentiviral production was performed as previously described^42,76^. Briefly, 293T cells were co-transfected with pCW107 or pLX303, pVSVg and pSPAX2 lentiviral packaging plasmids. Media was changed the next day to DMEM supplemented with 30% FBS. Two days later, media was collected and filtered through a 0.45 μm filter. Cells to be infected were seeded at a density of 500,000 cells per well in 6-well dishes or 100,000 cells per well in 24-well plates and spin-infected at 1200 x*g* the following day with 100–1000 µl of the aforementioned viral supernatant and 8 µg/ml hexadimethrine bromide (Sigma-Aldrich). Stable populations were selected in 1–2 µg/ml puromycin.

### CTKv2 screen

293T cells were independently infected with lentivirus derived from each of the 74 individual lentiviral constructs of the CTKv2 library, with each round of infections including a construct expressing luciferase as a control. Cells were passaged in puromycin-supplemented media, after which puromycin-resistant populations were frozen in 90% FBS and 10% DMSO freezing media. To perform arrayed screening of the CTKv2-transduced lines, 5–20 of these cell lines were thawed, seeded at 80,000 cells/well in 24-well plates, and transiently transfected with the indicated dual reporter constructs using the FuGENE 6 reagent according the manufacture’s protocol (Promega). Two days later, cells were subjected to FACS analysis to calculate the mean fluorescence intensity (MFI).

### FACS

Cells transfected with reporter constructs were analyzed with a BD Fortessa X-20 simultaneously assessing fluorescence emitted after laser excitation at 466 nm (GFP) and 561 nm (mCherry). Initial experiments established GFP and mCherry fluorescence parameters with single color and double negative controls. Data analysis was performed in Flowjo. mCherry-positive cells transfected with pcDNA3.1 + mCherry^com^:GFP^rare^ or GFP-positive cells transfected with pcDNA3.1 + mCherry^rare^:GFP^com^ were binned and assessed for mean mCherry and GFP fluorescence. For the CTKv2 screen, mean mCherry and GFP fluorescence were normalized to a parallel luciferase transduced control to generate a normalized value for reporter fluorescence.

### Immunoblot

To examine the expression of GFP constructs with different codon usage, 1 × 10^6^ 293 cells were seeded into each well of 6-well plates and were transfected the next day with pcDNA3.1 + GFP vector (0.6 µg) and pLX303Luciferase/BRAF^V600E^ vector (0.6 µg) using FuGENE 6 (Promega) according to the manufacturer's instructions. Two days later, transfected cells were lysed and assayed by immunoblot as described below. To compare the expression of GAG and RAS constructs, 1 × 10^6^ 293 cells were seeded into each well of 6-well plates and were transfected the next day with pcDNA3.1 + GAG/RAS (0.6 µg) and pLX303Luciferase/BRAF^V600E^ vector (0.6 µg) using FuGENE 6 (Promega). 36 h later, culture media were replaced with normal media or media containing 300 nM SCH772984 (Selleck Chemicals, S7101). After an additional day, cells were lysed and assayed by immunoblot as described below. ERK1/2 pathway drug studies were performed by stable infection of 293T cells with indicated cancer toolkit v2 constructs^42^ (pCW107BRAF^V600E^ or Luciferase), transfection of dual-reporter vectors (0.6 µg in each well of a 6-well plate, as above), and treatment of cells immediately following transfection with vehicle (DMSO; diluted 1:1000 v/v; Sigma-Aldrich; 472301), LY3009120 (Selleck Chemicals; S7842, 100 nM), Vemurafenib (Chemietek; CT-P4032, 300 nM), Trametinib (Chemietek; CT-GSK212, 50 nM), or SCH772984 (Selleck Chemicals; S7101, 100 nM). Two days later cells were collected for immunoblot and flow cytometry measurements of GFP and mCherry fluorescence. For all immunoblot analysis, cells were lysed in 5 mM EDTA, 50 mM Tris–HCl pH 8.0, 150 mM NaCl, 1% NP-40, 0.5% Sodium deoxycholate, 0.1% SDS, 1 mM NaF, 1 mM Na_3_VO_4_, and protease inhibitors (Roche, 11836170001). Protein concentration was measured using BCA kit (Thermo Fisher Scientific; 23225). Equal levels of protein lysate were resolved by SDS-PAGE and transferred onto PVDF membranes and probed with FLAG (Sigma-Aldrich F1804; 1:1000), GFP (Santa Cruz Biotechnology sc-9996; 1:1000), Phospho-p44/42 MAPK (Erk1/2) (Thr202/Tyr204) (Cell Signaling Technology 4376; 1:1000), p44/42 MAPK (Erk1/2) (Cell Signaling Technology 4695; 1:2000), Phospho-MEK1/2 (Ser217/221) (Cell Signaling Technology 9154; 1:1000), MEK1/2 (Cell Signaling Technology 9122; 1:1000), ß-Actin (Sigma-Aldrich A2228; 1:4000), ß-Tubulin (Sigma-Aldrich T5201; 1:2000) antibody. For quantification of immunoblots, optical density of target band was quantified using Image Lab Software (Bio-Rad) from images taken by Gel Doc XR + Imager (Bio-Rad) at exposure times when the signal intensity was not saturated. Images of uncut immunoblots are included in Supplementary Fig. 5.

### AHA-labelling

For AHA-labeling of GFP and mCherry protein, 293T cells (50% confluent) in 10 cm plate were transfected with 3 μg pcDNA3.1 + GFP^rare^:mCherry^com^, pcDNA3.1 + GFP^com^:mCherry^rare^, or pcDNA3.1 + empty vector and 3 μg pLX303 luciferase or pLX303 BRAF^V600E^. Media was changed 12 h later. One day later, the plates were methionine depleted for 30 min using DMEM SILAC media (AthenaES, 0420) reconstituted with arginine (AthenaES, 0416), lysine (AthenaES, 0417), leucine (AthenaES, 0418) and 10% dialyzed FBS (Sigma-Aldrich, F0392-100ML). AHA (Click Chemistry Tools, 1066-100) or methionine (AthenaES, 0419) was then added to media to 50 μM and cells were harvest 120 min later, or 30, 60 and 120 min later for time course experiment. Cells were collected in PBS. 10% of the cells were pelleted for RNA extraction while the rest were pelleted for protein extraction. For AHA-labeling of global proteins, 293T cells were transfected with pLX303 luciferase or pLX303 BRAF^V600E^. Two days later, the media was changed to methionine-free media, and 30 min later, 200 μM of AHA was added to media and cells were harvest 10, 20 and 40 min later.

### Immunoprecipitation and detection of AHA-labelled proteins

Cells were lysed in 5 mM EDTA, 50 mM Tris HCl pH 8.0, 150 mM NaCl, 1% NP-40, 0.5% Sodium deoxycholate, 0.1% SDS, 1 mM NaF, 1 mM Na_3_VO_4_, and protease inhibitors (Roche, 11836170001) for 30 min with end-to-end incubation at 4 °C. Protein concentration was measured using BCA kit (Thermo Fisher Scientific, 23225). Protein lysate was incubated with GFP (Santa Cruz Biotechnology, sc-9996, 1 μg per 500 μg total protein), mCherry (Novus Biologicals, NBP1-96752, 2.5 μg per 500 μg total protein), or IgG (Santa Cruz Biotechnology, sc-2025, 1 μg per 500 μg total protein) for 4 h to overnight by end-to-end rotation at 4 °C. Dynabeads Protein G (Thermo Fisher Scientific, 10003D) was washed twice in PBS, added to the lysate and antibody solution (25 μl per 500 μg total protein), and incubated for 20 min at room temperature. The beads were pelleted using magnetic separation rack (NEB, S1509S) and washed three times in wash buffer (PBS, 0.05% Triton X-100). The eluate was separated from the beads by heating at 70 °C for 10 min in elution buffer [100 m M TrisHCl pH 8.0, 1% SDS, and protease inhibitors (Roche, 11836170001)] followed by magnetic separation. Eluted protein was incubated with 10 mM iodoacetamide (Thermo Fisher Scientific, A39271) in dark for 30 min at room temperature and then with 0.1 mM Sulfo-DBCO-biotin (Sigma-Aldrich, 760706-5MG) for 4 h to overnight in dark for the click reaction between AHA and DBCO-biotin. Samples were then resolved by SDS-PAGE and immunoblotted for Biotin (Cell Signaling Technology, 5571; 1:1000), GFP (Santa Cruz Biotechnology, sc-9996; 1:1000) or mCherry (Novus Biologicals, NBP1-96752; 1:2000). 5% of input cell lysate was immunoblotted for GFP (Santa Cruz Biotechnology, sc-9996; 1:1000), mCherry (Novus Biologicals, NBP1-96752; 1:2000), Phospho-p44/42 MAPK (Erk1/2) (Thr202/Tyr204) (Cell Signaling Technology, 4376; 1:1000), p44/42 MAPK (Erk1/2) (Cell Signaling Technology, 4695; 1:2000), and ß-Tubulin (Sigma-Aldrich, T5201; 1:2000). To detect globally AHA-labeled proteins, 30 μg of total cell lysate was alkylated with 10 mM iodoacetamide, incubated with sulfo-DBCO-biotin (Sigma-Aldrich, 760706-5MG), resolved by SDS-PAGE and immunoblotted with an anti-biotin antibody (Sigma-Aldrich, SAB4200680) as above. Immunoblotting and Coomassie staining were performed in parallel to validate equal protein loading.

### qRT-PCR

293T cells were seeded in 6 well plate at 1 million cells per well. Two days later, cells were transfected with 250 ng of pcDNA3.1 + GFP^rare^:mCherry^com^, 250 ng pcDNA3.1 + GFP^com^:mCherry^rare^, and 500 ng of pLX303Luciferase or pLX303BRAF^V600E^. Media was changed after 12 h and cells were harvested for RNA extraction after 36 h. RNA was extracted using the TRIzol reagent (Thermo Fisher Scientific, 15596026), processed with TURBO DNA-free Kit (Thermo Fisher Scientific, AM1907), and converted to cDNA using iScript cDNA Synthesis Kit (Bio-Rad, 1708890). qPCR reactions were performed using iTaq Universal SYBR Green Supermix (Bio-Rad, 1725120) and CFX384 touch real-time PCR detection system (Bio-Rad). Primers sequences for qPCR are: GFP^rare^ F: 5′-ACATCATGGCAGACAAACCA-3′; GFP^rare^ R: 5′-AAAGGGCAGATTGTGTGGAC-3′; mCherry^com^ F: 5′-CACTACGACGCTGAGGTCAA-3′; mCherry^com^ R: 5′-CTCGTTGTGGGAAAGGATGT-3′; GFP^com^ F: 5′-GGCACAAGCTGGAGTACAAC-3′; GFP^com^ R: 5′-CGATGTTGTGGCGGATCTTG-3′; mCherry^rare^ F: 5′-GACGGAGGAGTTGTTACAGT-3′; mCherry^rare^ R: 5′-CTGCATAACAGGTCCATCCG-3′; Actin F: 5′-AACCGCGAGAAGATGACCC-3′; Actin R: 5′-ATCACGATGCCAGTGGTACG-3′.

### Statistical analysis

Data are shown as bar graphs mean ± SEM unless otherwise indicated. Two-tailed t-test was used for comparison of two groups. One-way or two-way analysis of variance (ANOVA) followed by Sidak's multiple comparisons test was used for comparison of more than two groups. *P* values ≥ 0.05 were considered not significant; *P < 0.05, **P < 0.01, ***P < 0.001, and ****P < 0.0001 were considered significant thresholds.

## Supplementary Information

Supplementary Information.

## Data Availability

All plasmids and all other data supporting the findings of this study are available from the corresponding author on reasonable request.
