# Supplementary material for: Expression of transgenes enriched in rare codons is enhanced by the MAPK pathway
Source: Sci Rep. 2020 Dec 17;10:22166. doi: 10.1038/s41598-020-78453-5 (PMC7746698; doi:10.1038/s41598-020-78453-5)
Supplement: Supplementary file 1 — Supplementary Information. [file 41598_2020_78453_MOESM1_ESM.pdf]

Supplementary Information

# Expression of mammalian transgenes enriched in rare codons is enhanced by the MAPK pathway

**Jackson Peterson[1,#], Siqi Li[1,#], Erin Kaltenbrun[1], Ozgun Erdogan[1], and Christopher M. Counter[1,2,*]**

[1]Department of Pharmacology & Cancer Biology, Duke University Medical Center, Durham NC, 27710.
[2]Department of Radiation Oncology, Duke University Medical Center, Durham NC, 27710.
[#]These authors contributed equally: Jackson Peterson, Siqi Li.
*email: count004@mc.duke.edu

Supplementary Figure 1. cDNAs.
Supplementary Figure 2. A reporter to characterize the expression of rare-codon enriched transcripts.
Supplementary Figure 3. MAPK pathway enhances the expression of rare-codon enriched transcripts.
Supplementary Figure 4. MAPK pathway enhances the translation of rare-codon enriched transcripts.
Supplementary Figure 5. Uncropped immunoblots shown in figures.
Supplementary Table 1. Summary of the Cancer Toolkit (CTK) lentiviral library screen.

**a**

cDNA: $GFP^{rare}$
CAI: 0.729
Source: gene synthesis

5'ATGGTGAGTAAAGGAGAAGAACTTTTCACTGGAGTTGTCCCAATTCTTGTTGAATTAGATGGCGATGTTAATG
GGCAAAAATTCTCTGTCAGTGGAGAGGGTGAAGGTGATGCAACATACGGAAAACTTACCCTTAAATTTATTTGC
ACTACTGGGAAGCTACCTGTTCCATGGCCAACACTTGTCACTACTCTGACCTATGGTGTTCAATGCTTTTCAAGAT
ACCCAGATCATATGAAACAGCATGACTTTTTCAAGAGTGCCATGCCCGAAGGTTATGTACAGGAAAGAACTATA
TTTTACAAAGATGACGGGAACTACAAGACACGTGCTGAAGTCAAGTTTGAAGGTGATACCCTTGTTAATAGAAT
CGAGTTAAAAGGTATTGATTTTAAAGAAGATGGAAACATTCTTGGACACAAAATGGAATACAACTATAACTCAC
ATAATGTATACATCATGGCAGACAAACCAAAGAATGGAATCAAAGTTAACTTCAAAATTAGACACAACATTAAA
GATGGAAGCGTTCAATTAGCAGACCATTATCAACAAAATACTCCAATTGGCGATGGCCCTGTCCTTTTACCAGAC
AACCATTACCTGTCCACACAATCTGCCCTTTCCAAAGATCCCAACGAAAAGAGAGATCACATGATCCTTCTTGAG
TTTGTAACAGCTGCTGGGATTACACATGGCATGGATGAACTATACAAATAA

**b**

cDNA: $GFP^{com}$
CAI: 0.962
Source: pEGFP-c3; Clontech

ATGGTGAGCAAGGGCGAGGAGCTGTTCACCGGGGTGGTGCCCATCCTGGTCGAGCTGGACGGCGACGTAAAC
GGCCACAAGTTCAGCGTGTCCGGCGAGGGCGAGGGCGATGCCACCTACGGCAAGCTGACCCTGAAGTTCATCT
GCACCACCGGCAAGCTGCCCGTGCCCTGGCCCACCCTCGTGACCACCCTGACCTACGGCGTGCAGTGCTTCAGC
CGCTACCCCGACCACATGAAGCAGCACGACTTCTTCAAGTCCGCCATGCCCGAAGGCTACGTCCAGGAGCGCAC
CATCTTCTTCAAGGACGACGGCAACTACAAGACCCGCGCCGAGGTGAAGTTCGAGGGCGACACCCTGGTGAAC
CGCATCGAGCTGAAGGGCATCGACTTCAAGGAGGACGGCAACATCCTGGGGCACAAGCTGGAGTACAACTACA
ACAGCCACAACGTCTATATCATGGCCGACAAGCAGAAGAACGGCATCAAGGTGAACTTCAAGATCCGCCACAAC
ATCGAGGACGGCAGCGTGCAGCTCGCCGACCACTACCAGCAGAACACCCCCATCGGCGACGGCCCCGTGCTGC
TGCCCGACAACCACTACCTGAGCACCCAGTCCGCCCTGAGCAAAGACCCCAACGAGAAGCGCGATCACATGGTC
CTGCTGGAGTTCGTGACCGCCGCCGGGATCACTCTCGGCATGGACGAGCTGTACAAGTAA

**c**

cDNA: $mCherry^{com}$
CAI: 0.965
Source: mCherry2-C1 plasmid; Addgene 54563

5'ATGGTGAGCAAGGGCGAGGAGGATAACATGGCCATCATCAAGGAGTTCATGCGCTTCAAGGTGCACATGGA
GGGCTCCGTGAACGGCCACGAGTTCGAGATCGAGGGCGAGGGCGAGGGCCGCCCCTACGAGGGCACCCAGAC
CGCCAAGCTGAAGGTGACCAAGGGTGGCCCCCTGCCCTTCGCCTGGGACATCCTGTCCCCTCAGTTCATGTACG
GCTCCAAGGCCTACGTGAAGCACCCCGCCGACATCCCCGACTACTTGAAGCTGTCCTTCCCCGAGGGCTTCAATT
GGGAGCGCGTGATGAACTTCGAGGACGGCGGCGTGGTGACCGTGACCCAGGACTCCTCCCTGCAGGACGGCG
AGTTCATCTACAAGGTGAAGCTGCGCGGCACCAACTTCCCCTCCGACGGCCCCGTAATGCAGTGTCGTACCATG
GGCTGGGAGGCCTCCACTGAGCGGATGTACCCCGAGGACGGCGCCCTGAAGGGCGAGATCAAGCAGAGGCTG
AAGCTGAAGGACGGCGGCCACTACGACGCTGAGGTCAAGACCACCTACAAGGCCAAGAAGCCCGTGCAGCTGC
CCGGCGCCTACAACGTCGACATCAAGTTGGACATCCTTTCCCACAACGAGGACTACACCATCGTGGAACAGTAC
GAACGCGCCGAGGGCCGCCACTCCACCGGCGGCATGGACGAGCTGTACAAGTAA

**d**

cDNA: $mCherry^{rare}$
CAI: 0.731
Source: gene synthesis

5’ATGGTCTCAAAGGGTGAAGAAGATAACATGGCAATTATTAAAGAGTTTATGCGTTTCAAGGTGCATATGGAG
GGATCTGTCAATGGGCATGAGTTTGAAATTGAAGGTGAAGGAGAAGGCCGACCATATGAGGGAACACAAACC
GCAAAACTAAAGGTAACTAAAGGCGGACCATTACCATTCGCCTGGGACATCCTCTCTCCACAGTTCATGTATGGA
AGTAAAGCTTATGTTAAACATCCGGCAGATATACCAGATTATTTGAAACTTTCATTCCCGGAGGGTTTTAAGTGG
GAACGCGTAATGAATTTTGAAGACGGAGGAGTTGTTACAGTGACGCAAGACTCAAGCCTCCAAGATGGAGAAT
TTATTTATAAAGTCAAACTTCGAGGAACGAATTTCCCCTCGGATGGACCTGTTATGCAGAAGAAGACTATGGGA
TGGGAAGCTTCAAGTGAAAGAATGTACCCTGAAGACGGTGCTCTTAAGGGAGAGATTAAACAACGTCTTAAATT
GAAAGATGGAGGACATTACGATGCTGAGGTGAAGACAACTTACAAAGCCAAAAAACCAGTTCAGCTGCCAGGA
GCGTACAATGTTAATATTAAACTGGATATCACCTCCCACAACGAGGATTACACTATCGTTGAGCAATATGAAAGA
GCTGAAGGGCGGCACTCGACAGGTGGCATGGATGAATTGTATAAGTAA

**e**

cDNA: $GFP^{opt20}$
CAI: 0.806
Source: gene synthesis, rare codons for all leucine and glycine converted to common, converted nucleotides are marked by lower case

5’ATGGTGAGTAAAGGcGAAGAACTgTTCACTGGgGTTGTCCCAATTCTgGTTGAActgGATGGCGATGTTAATGG
cCAAAAATTCTCTGTCAGTGGcGAGGGcGAAGGcGATGCAACATACGGcAAACTgACCCTgAAATTTATTTGCACT
ACTGGcAAGCTgCCTGTTCCATGGCCAACACTcGTCACTACTCTGACCTATGGcGTTCAATGCTTTTCAAGATACC
CAGATCATATGAAACAGCATGACTTTTTCAAGAGTGCCATGCCCGAAGGcTATGTACAGGAAAGAACTATATTTT
ACAAAGATGACGGcAACTACAAGACACGTGCTGAAGTCAAGTTTGAAGGcGATACCCTgGTTAATAGAATCGAG
ctgAAAGGcATTGATTTTAAAGAAGATGGcAACATTCTgGGgCACAAAATGGAATACAACTATAACTCACATAATG
TATACATCATGGCAGACAAACCAAAGAATGGcATCAAAGTTAACTTCAAAATTAGACACAACATTAAAGATGGc
AGCGTTCAActcGCAGACCATTATCAACAAAATACTCCAATTGGCGATGGCCCTGTCCTgctgCCAGACAACCATTA
CCTGTCCACACAATCTGCCCTgTCCAAAGATCCCAACGAAAAGAGAGATCACATGATCCTgCTgGAGTTTGTAAC
AGCTGCTGGGATTACACATGGCATGGATGAACTgTACAAATAA

**f**

cDNA: $GFP^{opt40}$
CAI: 0.858
Source: gene synthesis, rare codons for all leucine, glycine, valine and lysine converted to common, converted nucleotides are marked by lower case

5’ATGGTGAGTAAgGGcGAAGAACTgTTCACTGGgGTgGTgCCAATTCTgGTcGAActgGATGGCGATGTaAATGGc
CAAAAgTTCTCTGTgAGTGGcGAGGGcGAAGGcGATGCAACATACGGcAAgCTgACCCTgAAgTTTATTTGCACTA
CTGGcAAGCTgCCTGTgCCATGGCCAACACTcGTgACTACTCTGACCTATGGcGTgCAATGCTTTTCAAGATACCCA
GATCATATGAAgCAGCATGACTTTTTCAAGAGTGCCATGCCCGAAGGcTATGTcCAGGAAAGAACTATATTTTAC
AAgGATGACGGcAACTACAAGACACGTGCTGAAGTgAAGTTTGAAGGcGATACCCTgGTgAATAGAATCGAGctg
AAgGGcATTGATTTTAAgGAAGATGGcAACATTCTgGGgCACAAgATGGAATACAACTATAACTCACATAATGTcT
ACATCATGGCAGACAAgCCAAAGAATGGcATCAAgGTgAACTTCAAgATTAGACACAACATTAAAGATGGcAGCG
TgCAActcGCAGACCATTATCAACAAAATACTCCAATTGGCGATGGCCCTGTgCTgctgCCAGACAACCATTACCTG

TCCACACAATCTGCCCTgTCCAAAGATCCCAACGAAAAGAGAGATCACATGATCCTgCTgGAGTTTGTgACAGCT
GCTGGGATTACACATGGCATGGATGAACTgTACAAgTAA

**g**

cDNA: $GFP^{opt60}$
CAI: 0.888
Source: gene synthesis, rare codons for all leucine, glycine, valine, lysine, threonine, aspartic acid, and glutamic acid converted to common, converted nucleotides are marked by lower case

5'ATGGTGAGTAAgGGcGAgGAgCTgTTCACcGGgGTgGTgCCAATTCTgGTcGAgctgGAcGGCGAcGTaAATGGcC
AAAAgTTCTCTGTgAGTGGcGAGGGcGAgGGcGATGCAACcTACGGcAAgCTgACCCTgAAgTTTATTTGCACcACc
GGcAAGCTgCCTGTgCCATGGCCAACcCTcGTgACcACcCTGACCTATGGcGTgCAATGCTTTTCAAGATACCCAGA
cCATATGAAgCAGCATGACTTTTTCAAGAGTGCCATGCCCGAAGGcTATGTcCAGGAgAGAACcATATTTTACAAg
GAcGACGGcAACTACAAGACcCGTGCTGAgGTgAAGTTTGAgGGcGAcACCCTgGTgAATAGAATCGAGctgAAgG
GcATTGAcTTTAAgGAgGAcGGcAACATTCTgGGgCACAAgATGGAgTACAACTATAACTCACATAATGTcTACATC
ATGGCAGACAAgCCAAAGAATGGcATCAAgGTgAACTTCAAgATTAGACACAACATTAAAGAcGGcAGCGTgCAA
ctcGCAGACCATTATCAACAAAATACcCCAATTGGCGAcGGCCCTGTgCTgctgCCAGACAACCATTACCTGTCCACc
CAATCTGCCCTgTCCAAAGAcCCCAACGAgAAGAGAGATCACATGATCCTgCTgGAGTTTGTgACcGCTGCTGGGA
TTACtCATGGCATGGAcGAgCTgTACAAgTAA

**h**

cDNA: $GFP^{opt80}$
CAI: 0.912
Source: gene synthesis, rare codons for all leucine, glycine, valine, lysine, threonine, aspartic acid, glutamic acid, isoleucine, serine and proline converted to common, converted nucleotides are marked by lower case

5'ATGGTGAGcAAgGGcGAgGAgCTgTTCACcGGgGTgGTgCCcATcCTgGTcGAgctgGAcGGCGAcGTaAATGGcC
AAAAgTTCagcGTgtccGGcGAGGGcGAgGGcGATGCAACcTACGGcAAgCTgACCCTgAAgTTTATcTGCACcACcG
GcAAGCTgCCcGTgCCcTGGCCcACcCTcGTgACcACcCTGACCTATGGcGTgCAATGCTTTagcAGATACCCcGAcCA
TATGAAgCAGCATGACTTTTTCAAGtccGCCATGCCCGAAGGcTATGTcCAGGAgAGAACcATcTTTTACAAgGAcG
ACGGcAACTACAAGACcCGTGCTGAgGTgAAGTTTGAgGGcGAcACCCTgGTgAATAGAATCGAGctgAAgGGcAT
cGAcTTTAAgGAgGAcGGcAACATcCTgGGgCACAAgATGGAgTACAACTATAACagcCATAATGTcTACATCATGG
CAGACAAgCCAAAGAATGGcATCAAgGTgAACTTCAAgATcAGACACAACATcAAAGAcGGcAGCGTgCAActcGC
AGACCATTATCAACAAAATACcCCcATcGGCGAcGGCCCcGTgCTgctgCCcGACAACCATTACCTGagCACcCAATC
cGCCCTgagCAAAGAcCCCAACGAgAAGAGAGATCACATGATCCTgCTgGAGTTTGTgACcGCTGCTGGGATcACtC
ATGGCATGGAcGAgCTgTACAAgTAA

**i**

cDNA: $GFP^{opt100}$
CAI: 0.960
Source: gene synthesis, rare codons for all leucine, glycine, valine, lysine, threonine, aspartic acid, glutamic acid, isoleucine, serine, proline, phenylalanine, alanine, glutamine, histidine, asparagine, tyrosine and arginine converted to common, converted nucleotides are marked by lower case

5'ATGGTGAGcAAgGGcGAgGAgCTgTTCACcGGgGTgGTgCCcATcCTgGTcGAgctgGAcGGCGAcGTaAAcGGcC
AAAAgTTCagcGTgtccGGcGAGGGcGAgGGcGATGCcACcTACGGcAAgCTgACCCTgAAgTTcATcTGCACcACcG

GcAAGCTgCCcGTgCCcTGGCCcACcCTcGTgACcACcCTGACCTAcGGcGTgCAgTGCTTcagccgcTACCCcGAcCAc
ATGAAgCAGCAcGACTTcTTCAAGtccGCCATGCCCGAAGGcTAcGTcCAGGAgcGcACcATcTTcTACAAgGAcGAC
GGcAACTACAAGACcCGcGCcGAgGTgAAGTTcGAgGGcGAcACCCTgGTgAAccGcATCGAGctgAAgGGcATcGA
cTTcAAgGAgGAcGGcAACATcCTgGGgCACAAgATGGAgTACAACTAcAACagcCAcAAcGTcTAtATCATGGCcGA
CAAgCCAAAGAAcGGcATCAAgGTgAACTTCAAgATccGcCACAACATcAAAGAcGGcAGCGTgCAgctcGCcGACC
AcTAcCAgCAgAAcACcCCcATcGGCGAcGGCCCcGTgCTgctgCCcGACAACCAcTACCTGagCACcCAgTCcGCCCTg
agCAAAGAcCCCAACGAgAAGcGcGATCACATGATCCTgCTgGAGTTcGTgACcGCcGCcGGGATcACtCATGGCAT
GGAcGAgCTgTACAAgTAA

## j

cDNA: FLAG-GAG
CAI: 0.759
Source: pGAG-EGFP

ATGGACTACAAGGACGACGATGACAAGATGGGTGCGAGAGCGTCAGTATTAAGCGGGGGAGAATTAGATCGA
TGGGAAAAAATTCGGTTAAGGCCAGGGGGAAAGAAGAAGTACAAGCTAAAGCACATCGTATGGGCAAGCAGG
GAGCTAGAACGATTCGCAGTTAATCCTGGCCTGTTAGAAACATCAGAAGGCTGTAGACAAATACTGGGACAGCT
ACAACCATCCCTTCAGACAGGATCAGAGGAGCTTCGATCACTATACAACACAGTAGCAACCCTCTATTGTGTGCA
CCAGCGGATCGAGATCAAGGACACCAAGGAAGCTTTAGACAAGATAGAGGAAGAGCAAAACAAGTCCAAGAA
GAAGGCCCAGCAGGCAGCAGCTGACACAGGACACAGCAATCAGGTCAGCCAAAATTACCCTATAGTGCAGAAC
ATCCAGGGGCAAATGGTACATCAGGCCATATCACCTAGAACTTTAAATGCATGGGTAAAAGTAGTAGAAGAGA
AGGCTTTCAGCCCAGAAGTGATACCCATGTTTTCAGCATTATCAGAAGGAGCCACCCCACAGGACCTGAACACG
ATGTTGAACACCGTGGGGGGACATCAAGCAGCCATGCAAATGTTAAAAGAGACCATCAATGAGGAAGCTGCAG
AATGGGATAGAGTGCATCCAGTGCATGCAGGGCCTATTGCACCAGGCCAGATGAGAGAACCAAGGGGAAGTG
ACATAGCAGGAACTACTAGTACCCTTCAGGAACAAATAGGATGGATGACAAATAATCCACCTATCCCAGTAGGA
GAGATCTACAAGAGGTGGATAATCCTGGGATTGAACAAGATCGTGAGGATGTATAGCCCTACCAGCATTCTGG
ACATAAGACAAGGACCAAAAGAACCCTTTAGAGACTATGTAGACCGGTTCTATAAAACTCTAAGAGCTGAGCAA
GCTTCACAGGAGGTAAAAAATTGGATGACAGAAACCTTGTTGGTCCAAAATGCGAACCCAGATTGTAAGACCAT
CCTGAAGGCTCTCGGCCCAGCGGCTACACTAGAAGAAATGATGACAGCATGTCAGGGAGTAGGAGGACCCGGC
CATAAGGCAAGAGTTTTGGCTGAAGCAATGAGCCAAGTAACAAATTCAGCTACCATAATGATGCAGAGAGGCA
ATTTTAGGAACCAAAGAAAGATTGTTAAGTGTTTCAATTGTGGCAAAGAAGGGCACACAGCCAGAAATTGCAG
GGCCCCTAGGAAAAAGGGCTGTTGGAAATGTGGAAAGGAAGGACACCAAATGAAAGATTGTACTGAGAGACA
GGCTAATTTTTTAGGGAAGATCTGGCCTTCCTACAAGGGAAGGCCAGGGAATTTTCTTCAGAGCAGACCAGAGC
CAACAGCCCCACCAGAAGAGAGCTTCAGGTCTGGGGTAGAGACAACAACTCCCCCTCAGAAGCAGGAGCCGAT
AGACAAGGAACTGTATCCTTTAACTTCCCTCAGATCACTCTTTGGCAACGACCCCTCGTCACAATAA

## k

cDNA: FLAG-GAG$^{com}$
CAI: 0.995
Source: gene synthesis, based on p96ZM651.8 Gag sequence (NIH AIDS Reagent Program; 8675) with codon-optimized amino acid substitutions to match the exact amino acid sequence of Gag$^{rare}$.

ATGGACTACAAGGACGACGATGACAAGATGGGCGCCCGCGCCAGCGTGCTGAGCGGCGGCGAGCTGGACCGG
TGGGAGAAGATCCGCCTGCGCCCCGGCGGCAAGAAGAAGTACAAGCTGAAGCACATCGTGTGGGCCAGCCGC
GAGCTGGAGCGCTTCGCCGTGAACCCCGGCCTGCTGGAGACCAGCGAGGGCTGCCGGCAGATCCTGGGCCAG
CTGCAGCCCAGCCTGCAGACCGGCAGCGAGGAGCTGCGCAGCCTGTACAACACCGTGGCCACCCTGTACTGCG
TGCACCAGCGGATCGAGATCAAGGACACCAAGGAGGCCCTGGACAAGATCGAGGAGGAGCAGAACAAGAGCA

AGAAGAAGGCCCAGCAGGCCGCCGCCGACACCGGCCACAGCAACCAGGTGAGCCAGAACTACCCCATCGTGCA
GAACATCCAGGGCCAGATGGTGCACCAGGCCATCAGCCCCCGCACCCTGAACGCCTGGGTGAAGGTGGTGGAG
GAGAAGGCCTTCAGCCCCGAGGTGATCCCCATGTTCAGCGCCCTGAGCGAGGGCGCCACCCCCCAGGACCTGA
ACACCATGCTGAACACCGTGGGCGGCCACCAGGCCGCCATGCAGATGCTGAAGGAGACCATCAACGAGGAGG
CCGCCGAGTGGGACCGCGTGCACCCCGTGCACGCCGGCCCCATCGCCCCCGGCCAGATGCGCGAGCCCCGCGG
CAGCGACATCGCCGGCACCACCAGCACCCTGCAGGAGCAGATCGGCTGGATGACCAACAACCCCCCCATCCCCG
TGGGCGAGATCTACAAGCGCTGGATCATCCTGGGCCTGAACAAGATCGTGCGCATGTACAGCCCCACCAGCATC
CTGGACATCCGGCAGGGCCCCAAGGAGCCCTTCCGCGACTACGTGGACCGCTTCTACAAGACCCTGCGCGCCGA
GCAGGCCAGCCAGGAGGTGAAGAACTGGATGACCGAGACCCTGCTGGTGCAGAACGCCAACCCCGACTGCAA
GACCATCCTGAAGGCCCTGGGCCCCGCCGCCACCCTGGAGGAGATGATGACCGCCTGCCAGGGCGTGGGCGGC
CCCGGCCACAAGGCCCGCGTGCTGGCCGAGGCCATGAGCCAGGTGACCAACAGCGCCACCATCATGATGCAGC
GGGGCAACTTCCGGAACCAGCGGAAGATCGTGAAGTGCTTCAACTGCGGCAAGGAGGGCCACACCGCCCGCA
ACTGCCGCGCCCCCCGCAAGAAGGGCTGCTGGAAGTGCGGCAAGGAGGGCCACCAGATGAAGGACTGCACCG
AGCGCCAGGCCAACTTCCTGGGCAAGATCTGGCCCAGCTACAAGGGCCGCCCCGGCAACTTCCTGCAGAGCCG
CCCCGAGCCCACCGCCCCCCCCGAGGAGAGCTTCCGCAGCGGCGTGGAGACCACCACCCCCCCCCAGAAGCAG
GAGCCCATCGACAAGGAGCTGTACCCCCTGACCAGCCTGCGGAGCCTGTTCGGCAACGACCCCAGCAGCCAGT
AA

## l

cDNA: FLAG-KRAS
CAI: 0.752
Source: Cloned from previously described pBabePuro-KRAS construct[22].
ATGGACTACAAAGACGATGACGACAAGACTGAATATAAACTTGTGGTAGTTGGAGCTGGTGGCGTAGGCAAGA
GTGCCTTGACGATACAGCTAATTCAGAATCATTTTGTGGACGAATATGATCCAACAATAGAGGATTCCTACAGG
AAGCAAGTAGTAATTGATGGAGAAACCTGTCTCTTGGATATTCTCGACACAGCAGGTCAAGAGGAGTACAGTGC
AATGAGGGACCAGTACATGAGGACTGGGGAGGGCTTTCTTTGTGTATTTGCCATAAATAATACTAAATCATTTG
AAGATATTCACCATTATAGAGAACAAATTAAAAGAGTTAAGGACTCTGAAGATGTACCTATGGTCCTAGTAGGA
AATAAATGTGATTTGCCTTCTAGAACAGTAGACACAAAACAGGCTCAGGACTTAGCAAGAAGTTATGGAATTCC
TTTTATTGAAACATCAGCAAAGACAAGACAGAGAGTGGAGGATGCTTTTTATACATTGGTGAGAGAGATCCGAC
AATACAGATTGAAAAAAATCAGCAAAGAAGAAAAGACTCCTGGCTGTGTGAAAATTAAAAAATGCATTATAATG
TAA

## m

cDNA: FLAG-HRAS
CAI: 0.90
Source: Cloned from previously described pBabePuro-HRAS construct[22].

ATGGACTACAAAGACGATGACGACAAGACGGAATATAAGCTGGTGGTGGTGGGCGCTGGAGGCGTGGGAAAG
AGTGCCCTGACCATCCAGCTGATCCAGAACCACTTTGTGGACGAATACGACCCCACTATAGAGGATTCCTACCG
GAAGCAGGTGGTCATTGATGGGGAGACGTGCCTGTTGGACATCCTGGATACCGCCGGCCAGGAGGAGTACAG
CGCCATGCGGGACCAGTACATGCGCACCGGGGAGGGCTTCCTGTGTGTGTTTGCCATCAACAACACCAAGTCTT
TTGAGGACATCCACCAGTACAGGGAGCAGATCAAACGGGTGAAGGACTCGGATGACGTGCCCATGGTGCTGGT
GGGGAACAAGTGTGACCTGGCTGCACGCACTGTGGAATCTCGGCAGGCTCAGGACCTCGCCCGAAGCTACGGC
ATCCCCTACATCGAGACCTCGGCCAAGACCCGGCAGGGAGTGGAGGATGCCTTCTACACGTTGGTGCGTGAGA
TCCGGCAGCACAAGCTGCGGAAGCTGAACCCTCCTGATGAGAGTGGCCCCGGCTGCATGAGCTGCAAGTGTGT
GCTCTCCTGA

**Supplementary Figure 1. cDNAs. (a-m)** Gene name, CAI, source, and nucleotide sequence of cDNAs used as reporters.

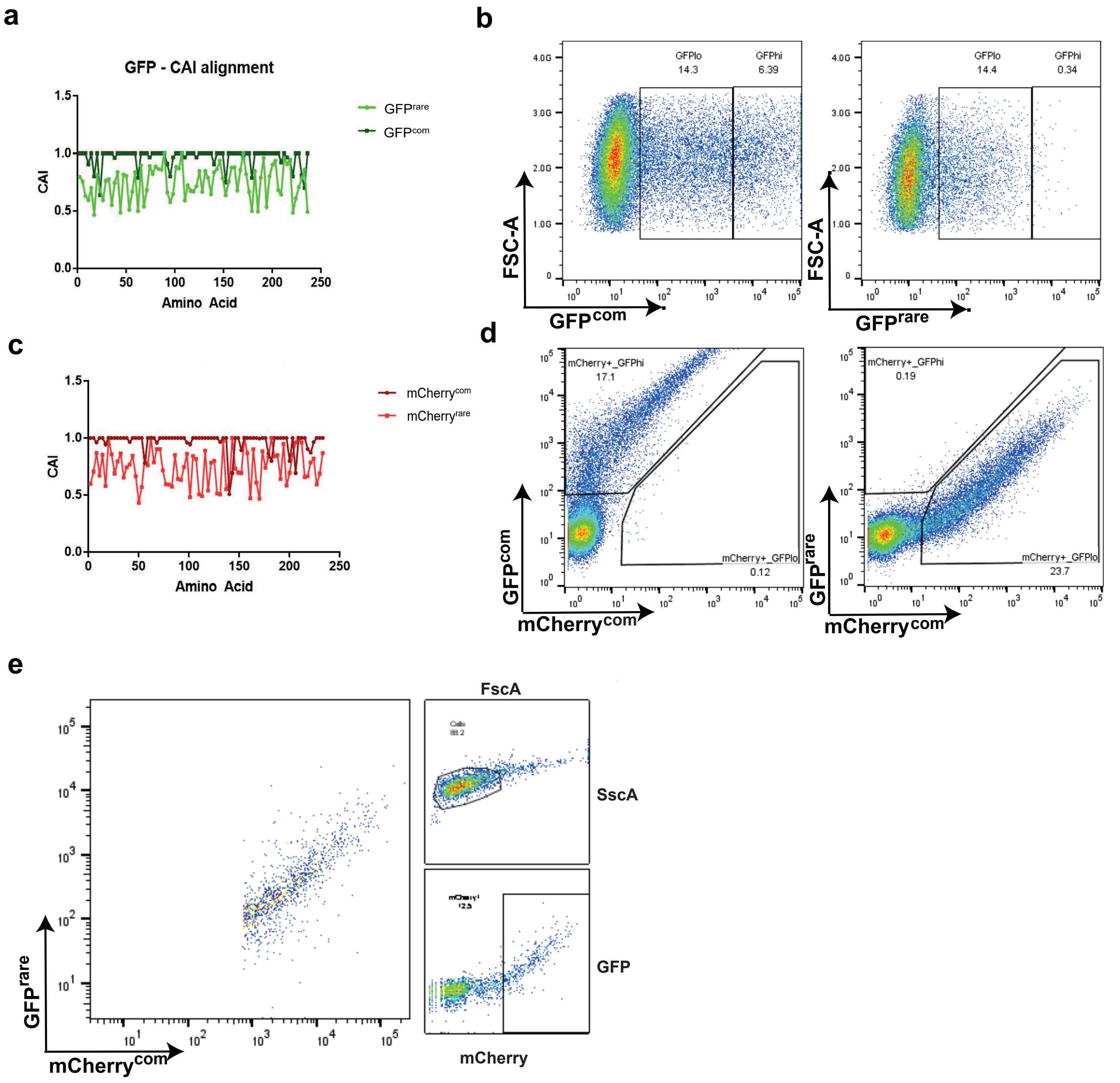


**Supplementary Figure 2. A reporter to characterize the expression of rare-codon enriched transcripts. (a,c)** Codon Adaptation Index (CAI) plotted as a function of amino acid residue for the indicated reporters. **(b,d)** Flow cytometry plots of single **(b)** and dual color **(d)** reporters with gates drawn to illustrate differences in the ability of each respective assay to exclude events from reporters using different codon usages. X axis: GFP intensity; Y axis: forward scatter area; GFPlo: GFP low; GFPhi: GFP high; Number indicates the percentage of highlighted population. **(e)** Flow cytometry plots demonstrating common gating scheme used to generate mean fluorescence intensities in this work; cells were gated according to forward and side scatter (FSC and SSC, respectively) and the MFI of positively transfected cells was calculated for each fluorescent reporter.

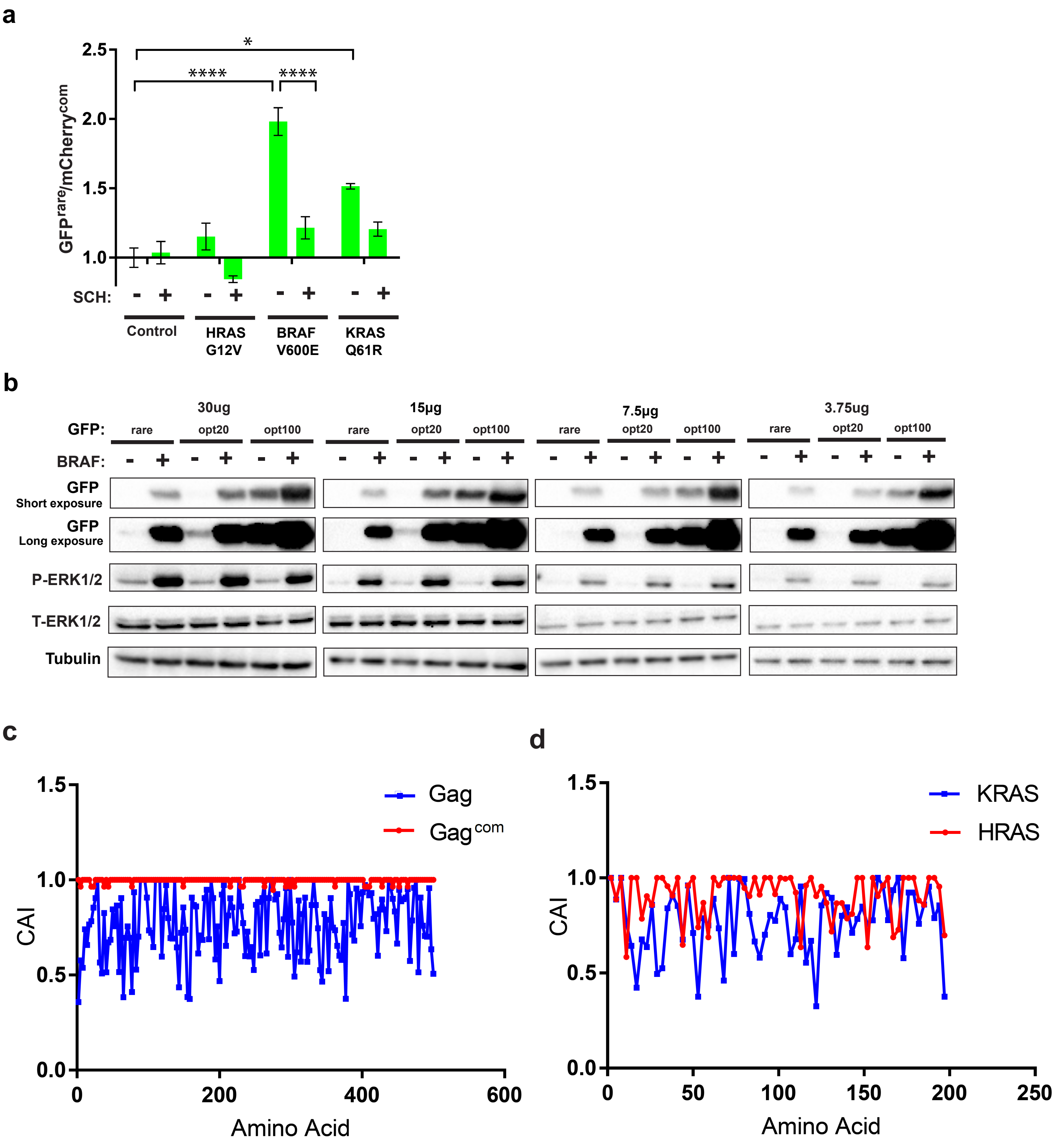


**Supplementary Figure 3. MAPK pathway enhances the expression of rare-codon enriched transcripts.** **(a)** Mean ± SEM of the ratio of GFP$^{rare}$ to mCherry$^{com}$ fluorescence of 293T cells stably infected with lentivirus encoding the indicated transgenes normalized to the fluorescence of parallel luciferase control (Ctrl) cells in the absence and presence of DMSO vehicle (-) or the SCH779284 ERK inhibitor (+) from three technical replicates. Two-way ANOVA with Sidak's multiple comparisons test: *$p < 0.05$ and ****$p < 0.0001$. **(b)** Expanded immunoblot for Fig. 3b. Blots separated by white space are from different parts of the same gel or from different gels at the same exposure in the same experiment. Specifically, 30µg and 15ug samples were loaded on one gel while 7.5ug and 3.75ug samples were loaded on a separate gel. **(c,d)** Codon Adaptation Index (CAI) plotted as a function of amino acid residue for the indicated reporters.

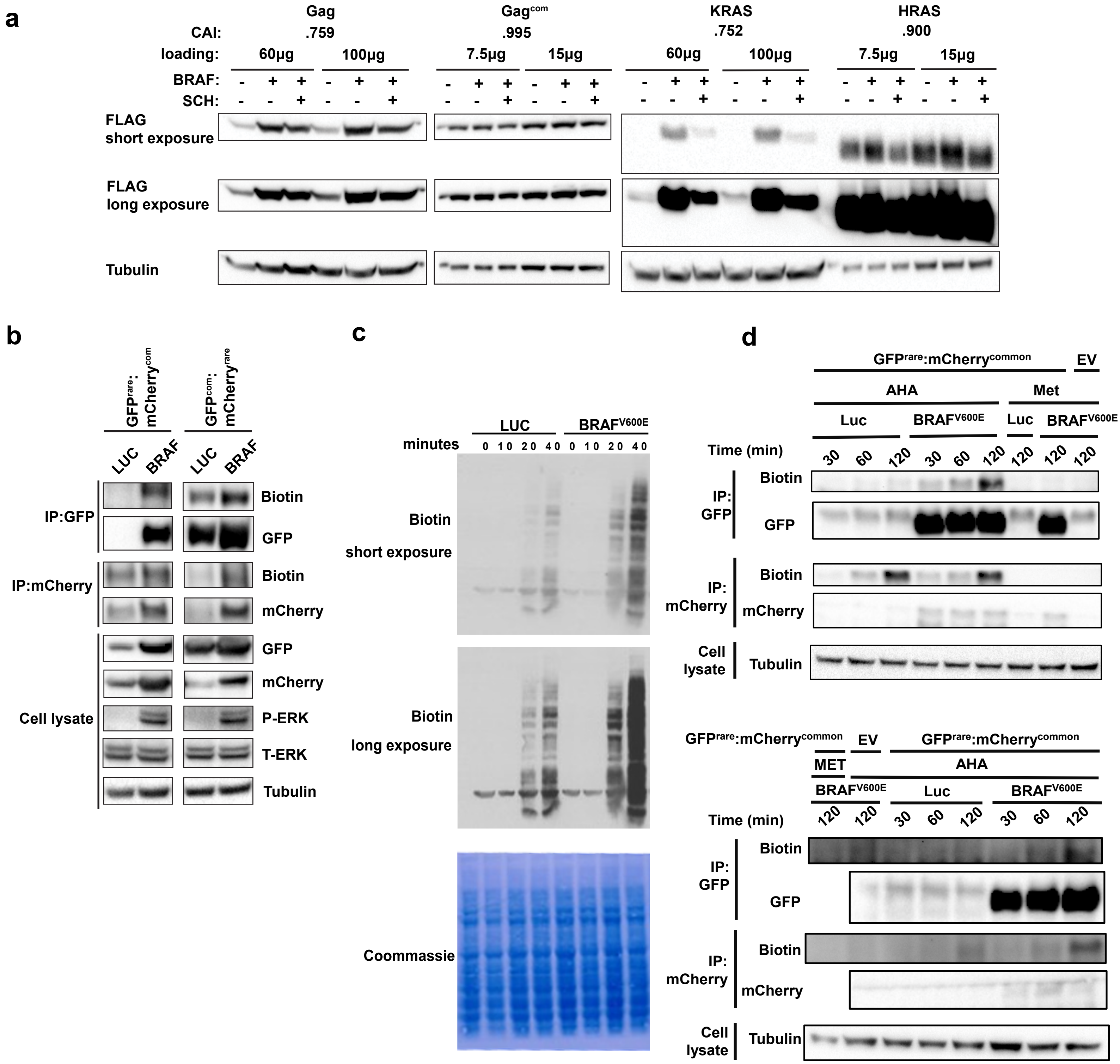


**Supplementary Figure 4. MAPK pathway enhances the translation of rare-codon enriched transcripts. (a)** Immunoblot analysis of FLAG-tagged Gag, Gag$^{com}$, KRAS, HRAS, and tubulin from 293 cells co-transfected with a vector encoding either luciferase control (-) or BRAF$^{V600E}$ (+) and the indicated FLAG-tagged proteins in the absence (-) or presence (+) of the ERK1/2 inhibitor SCH772984 (SCH). The lysate is the same as that used for figure 3c with variations in protein loading amount as indicated. Blots separated by white space are from different gels. **(b)** Biological replicate of figure 3d. Left and right blots (separated by white space) are from different parts of the same gel at different exposures, except for cell lysate: mCherry, P-ERK, T-ERK and Tubulin, which are different parts of the same gel at the same exposure. **(c)** Immunoblot analysis of whole cell lysates derived from 293T cells transiently expressing BRAF$^{V600E}$ (BRAF) or control luciferase (Luc) and labeled with AHA for the indicated times. Coommassie stain: control for protein loading. One of two biological replicates. **(d)** Replicate experiments of immunoblot analysis of AHA-biotin labeled mCherry or GFP in 293T cells transiently expressing mCherry$^{com}$ and GFP$^{rare}$ or mCherry$^{rare}$ and GFP$^{com}$ in the presence of BRAF$^{V600E}$ (BRAF) or control luciferase (Luc) and labeled with AHA for the indicated times. EV: empty vector, AHA: L-Azidohomoalanine, Met: methionine.

Fig 2b

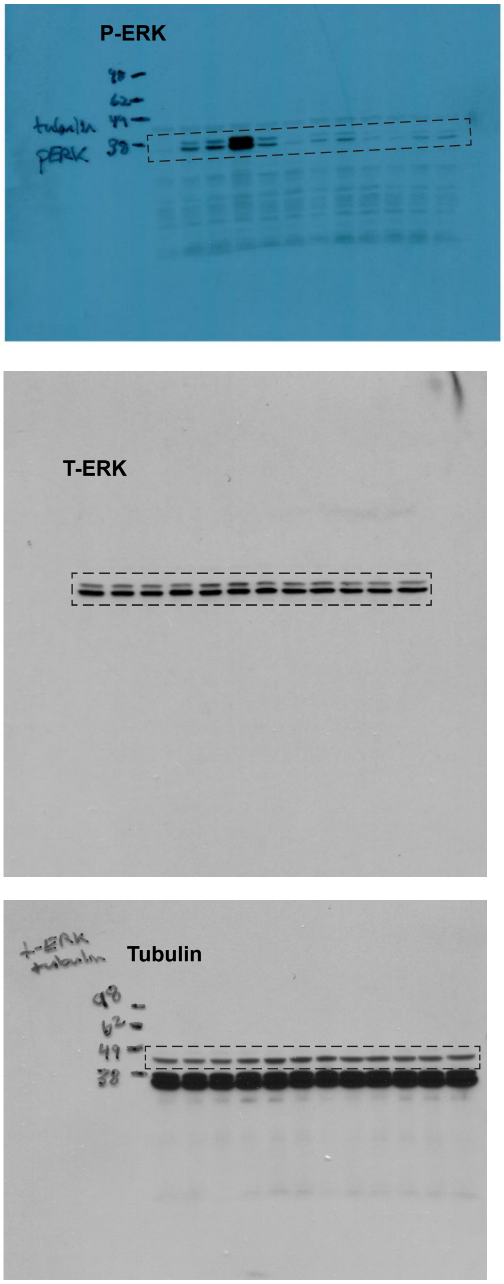


Fig 2c

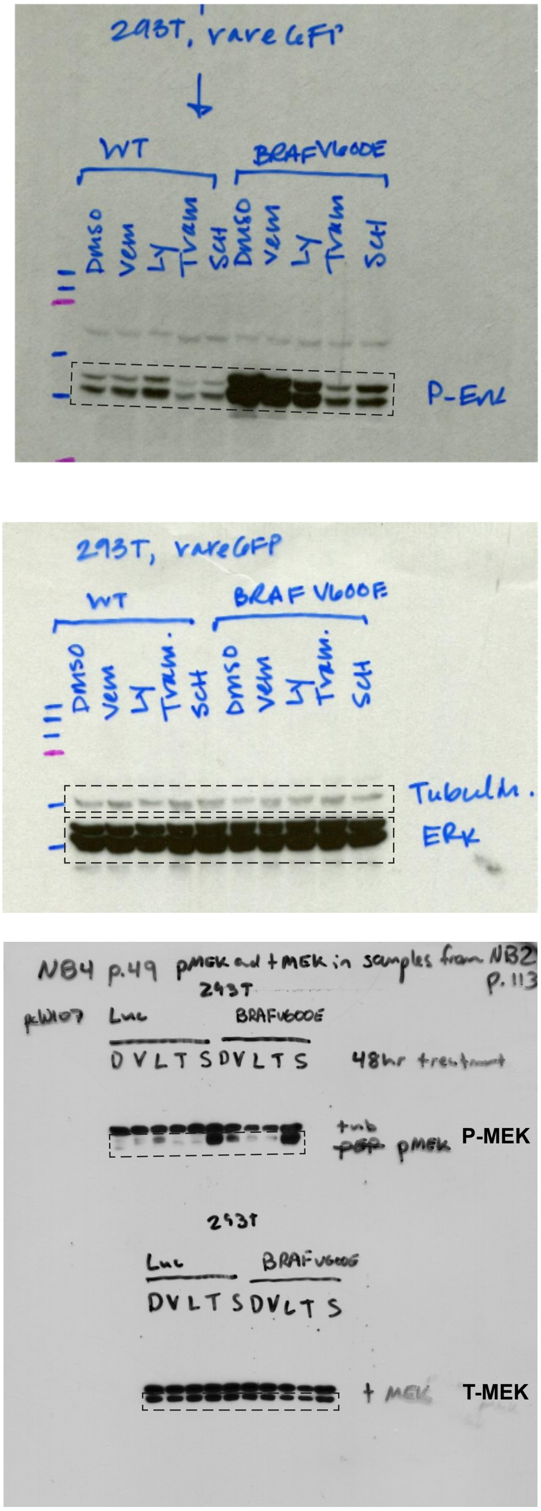


Fig 2d

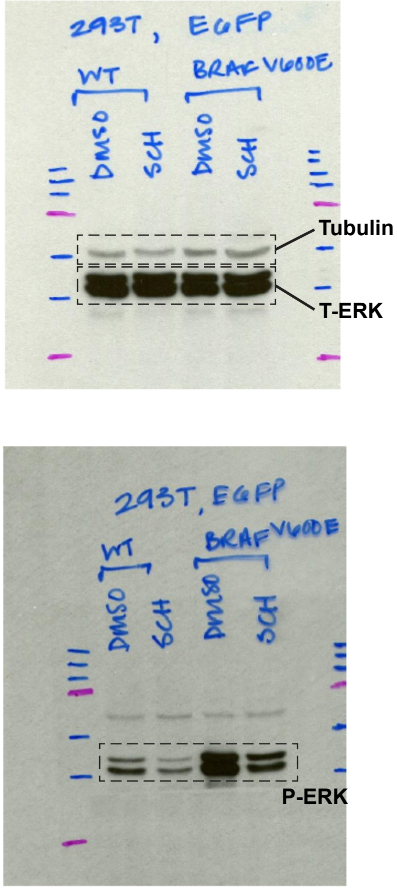


**Supplementary Figure 5. Uncropped blots.**

## Figure 3a

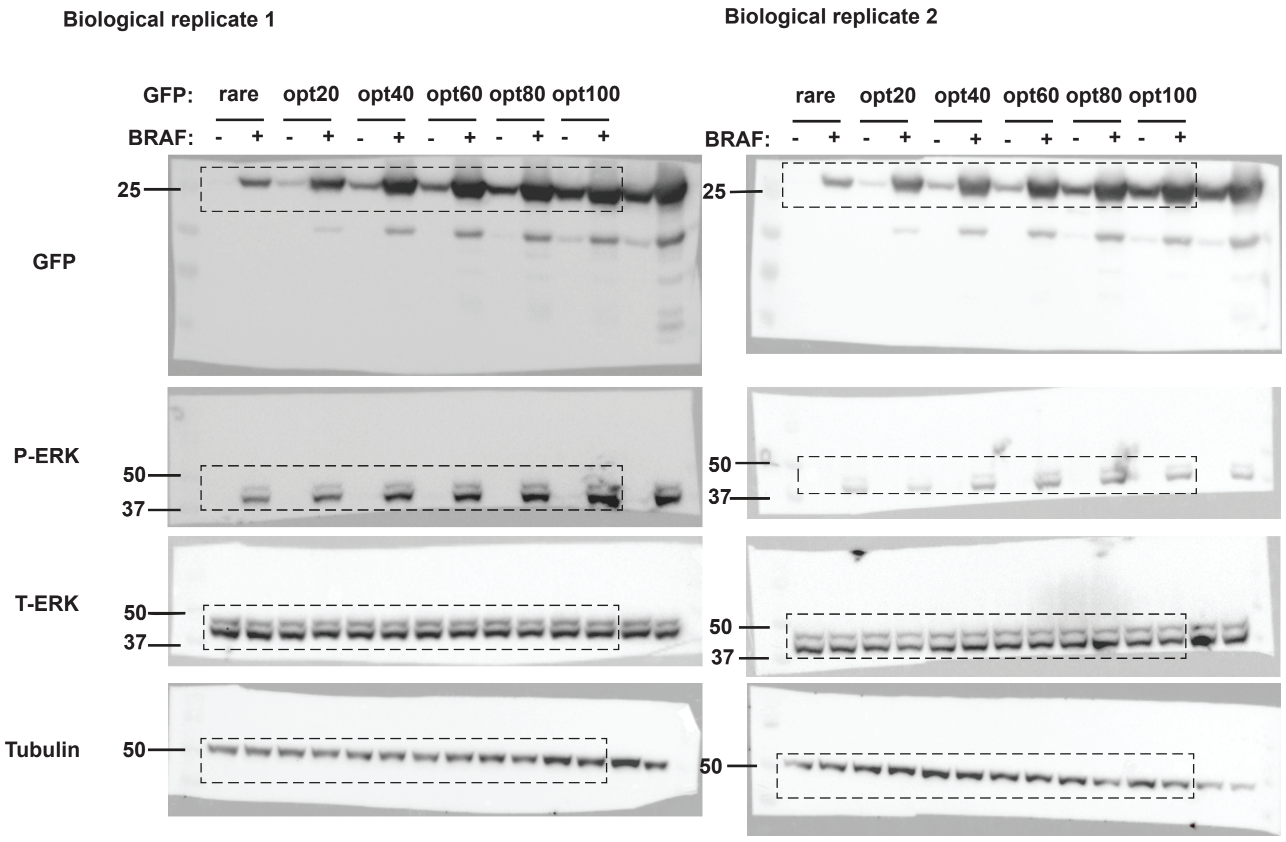


## Figure 3b (red box) and Supplementary Figure 3b (black box)

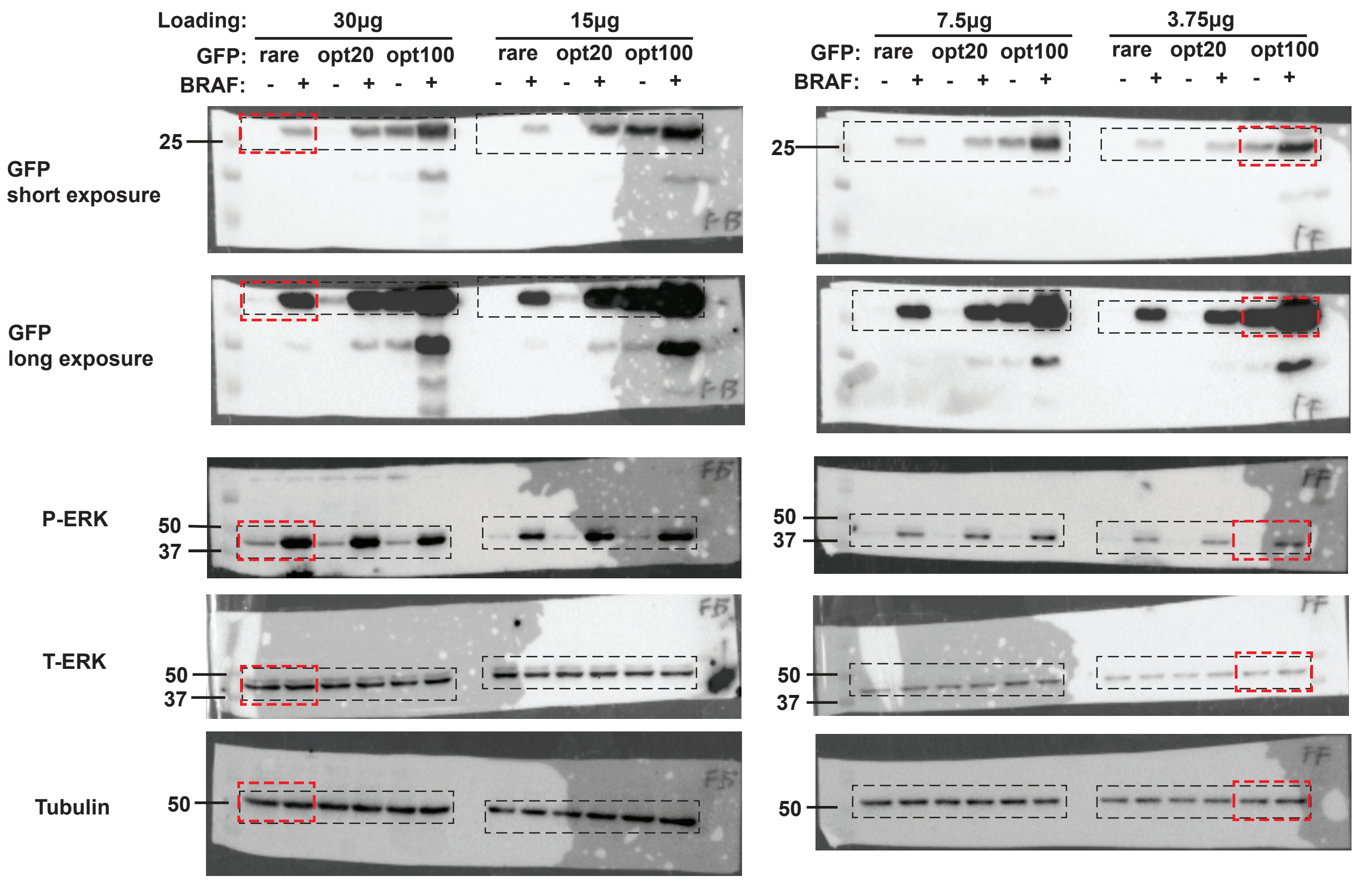


**Supplementary Figure 5, continued. Uncropped blots.**

**Figure 3c**

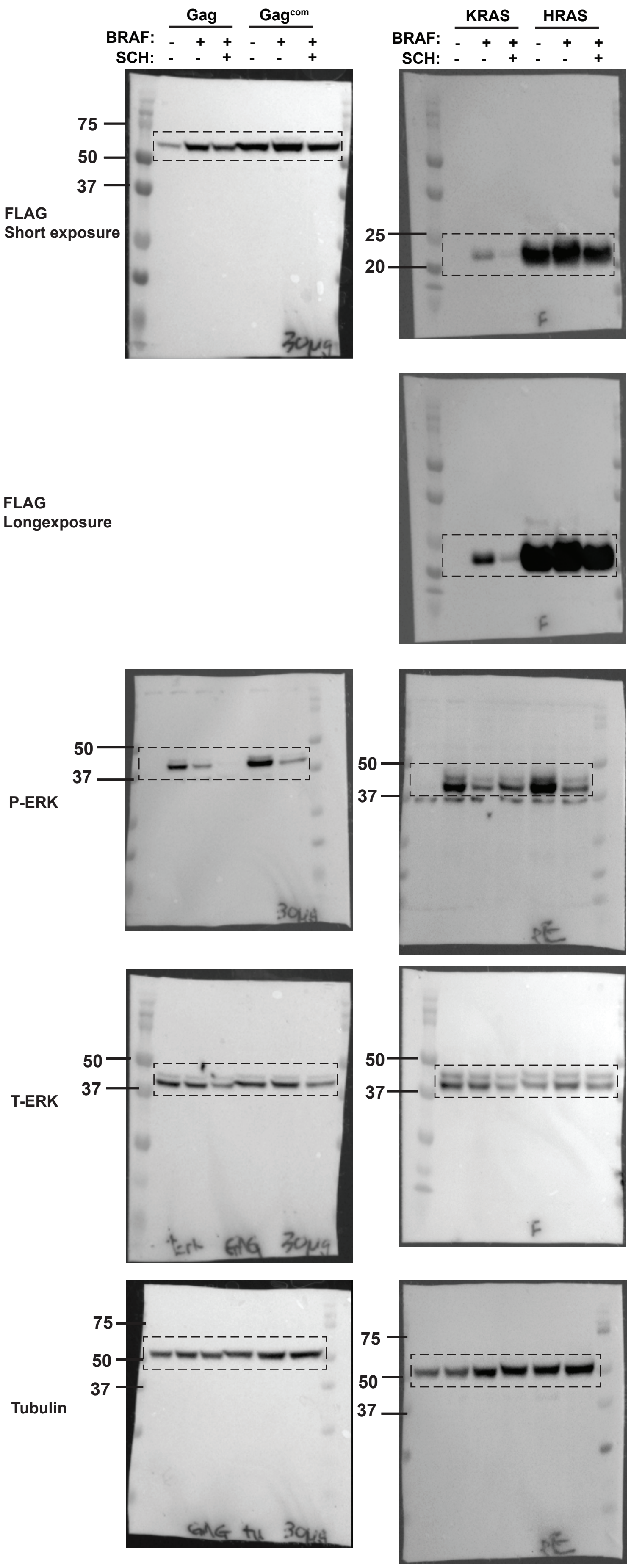


**Supplementary Figure 5, continued. Uncropped blots.**

Figure 3d

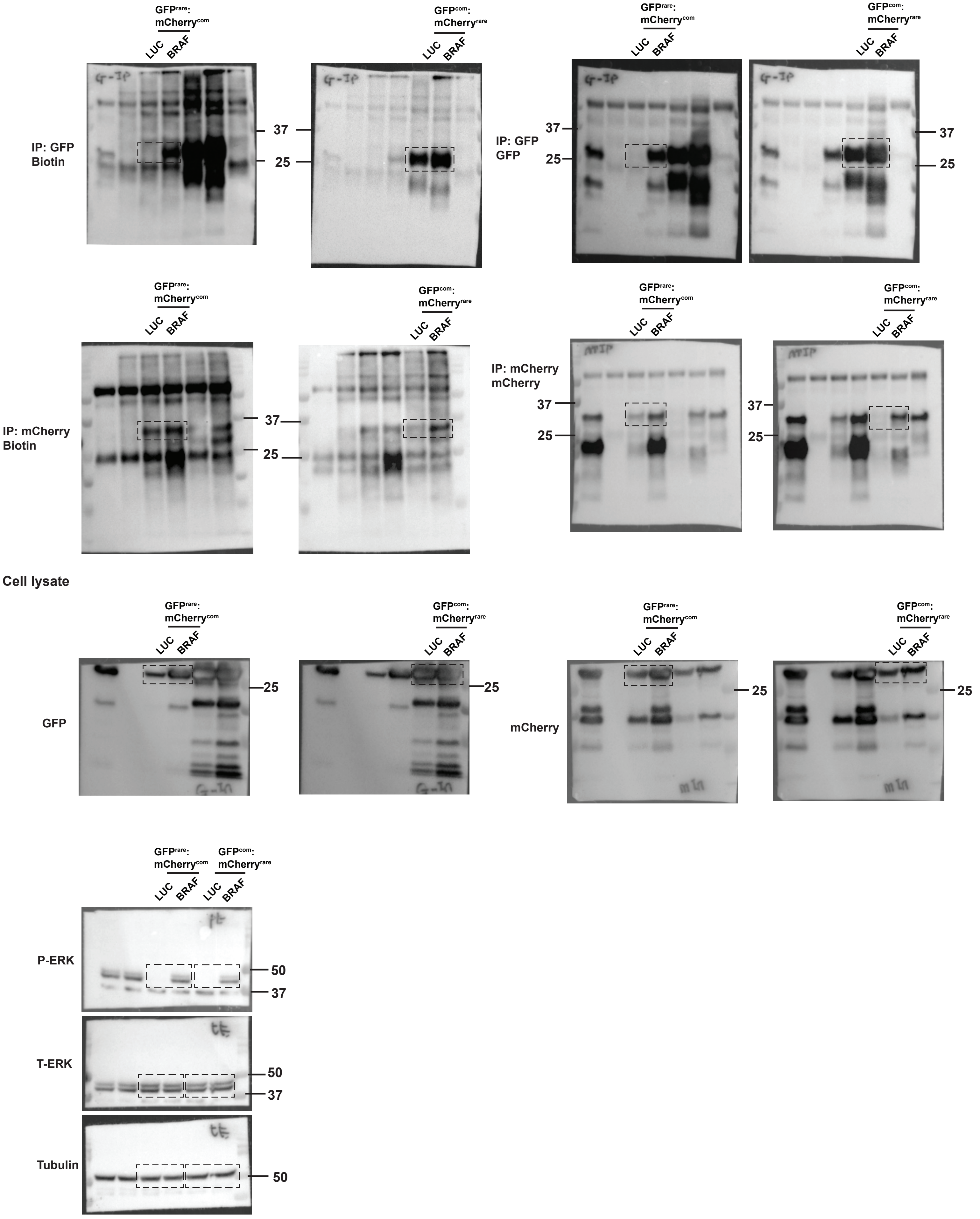


Supplementary Figure 5, continued. Uncropped blots.

## Supplementary Figure 4a

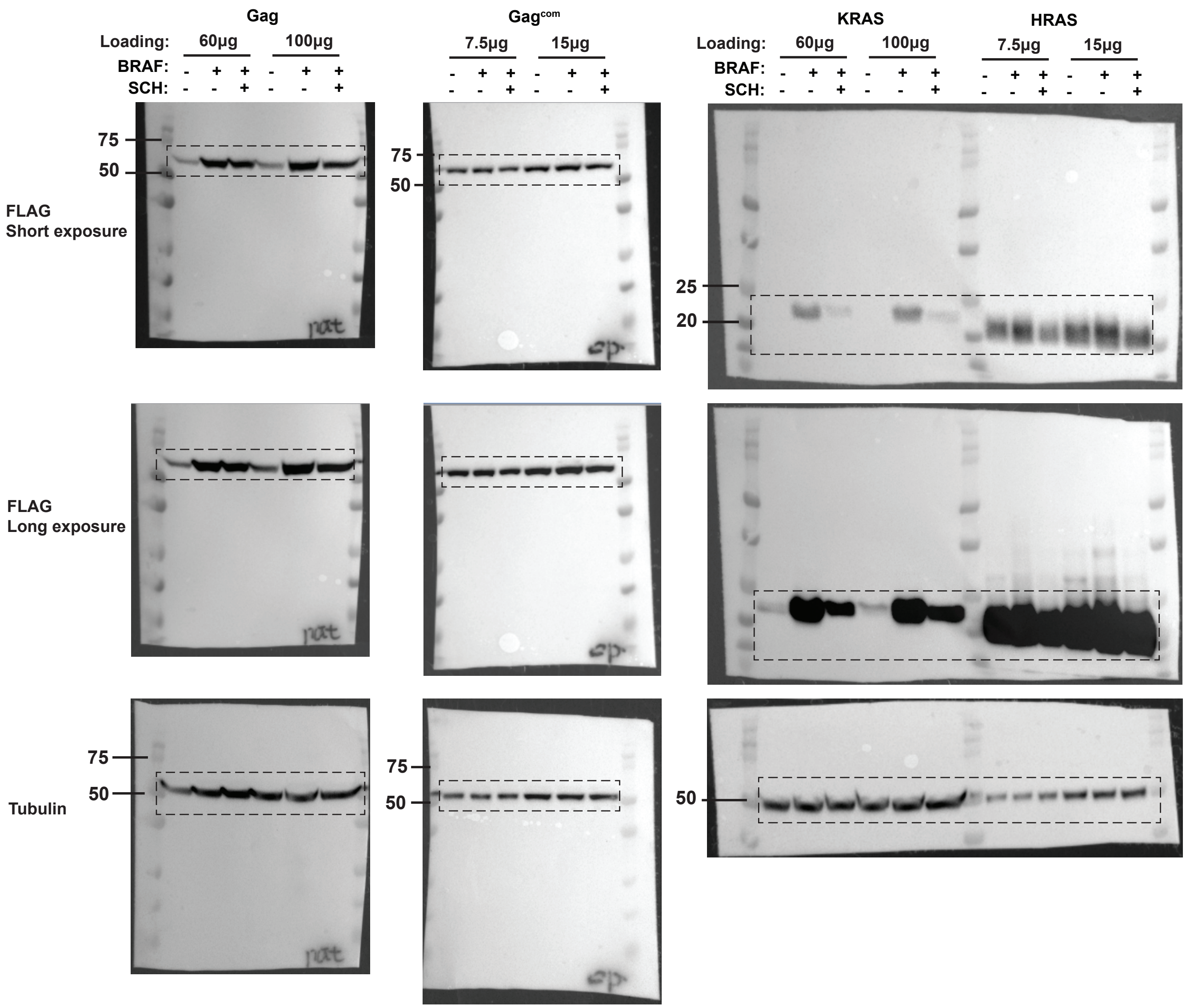


**Supplementary Figure 5, continued. Uncropped blots.**

## Supplementary Figure 4b

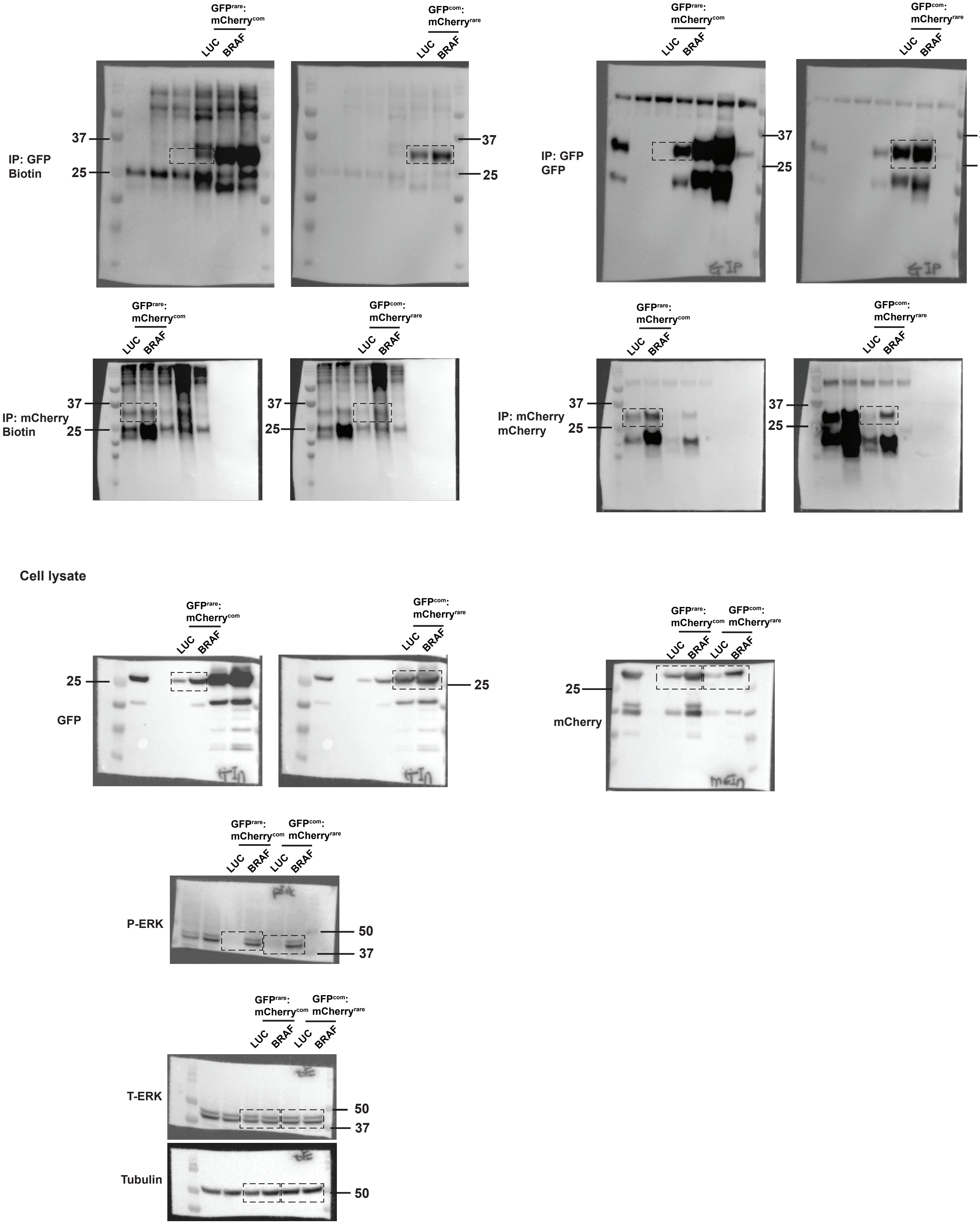


**Supplementary Figure 5, continued. Uncropped blots.**

**Supplementary Figure 4c** Uncropped immunoblots shown in figures.

**Supplementary Figure 4d top**

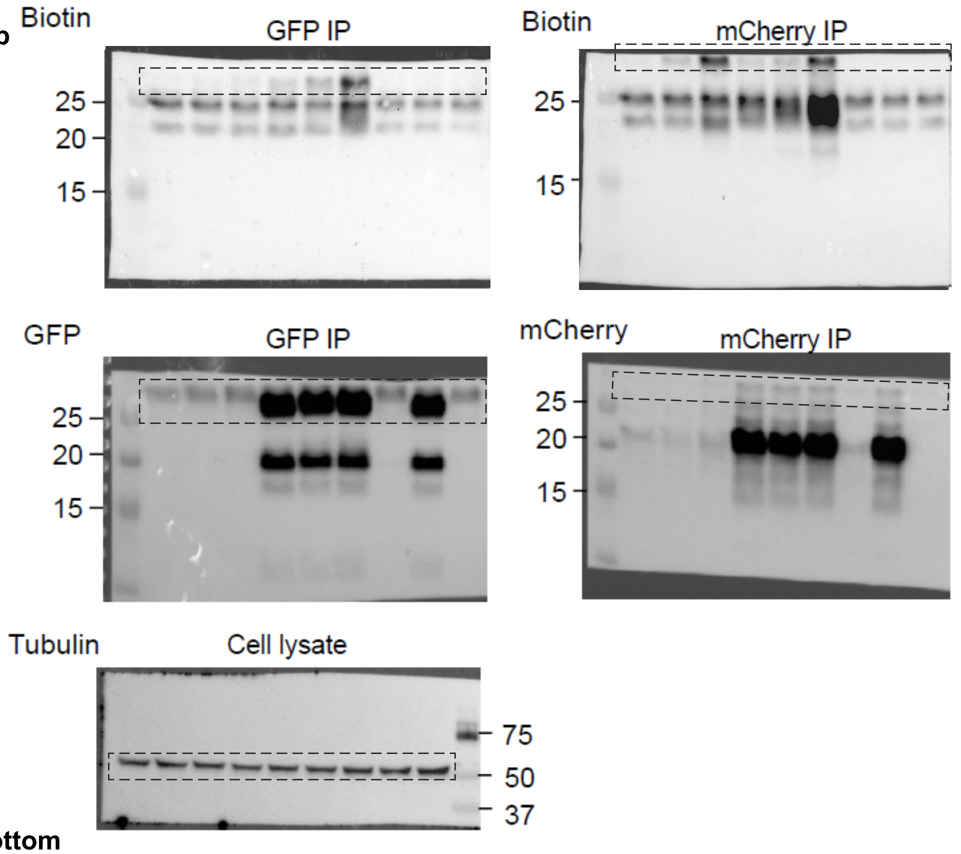


**Supplementary Figure 4d bottom**

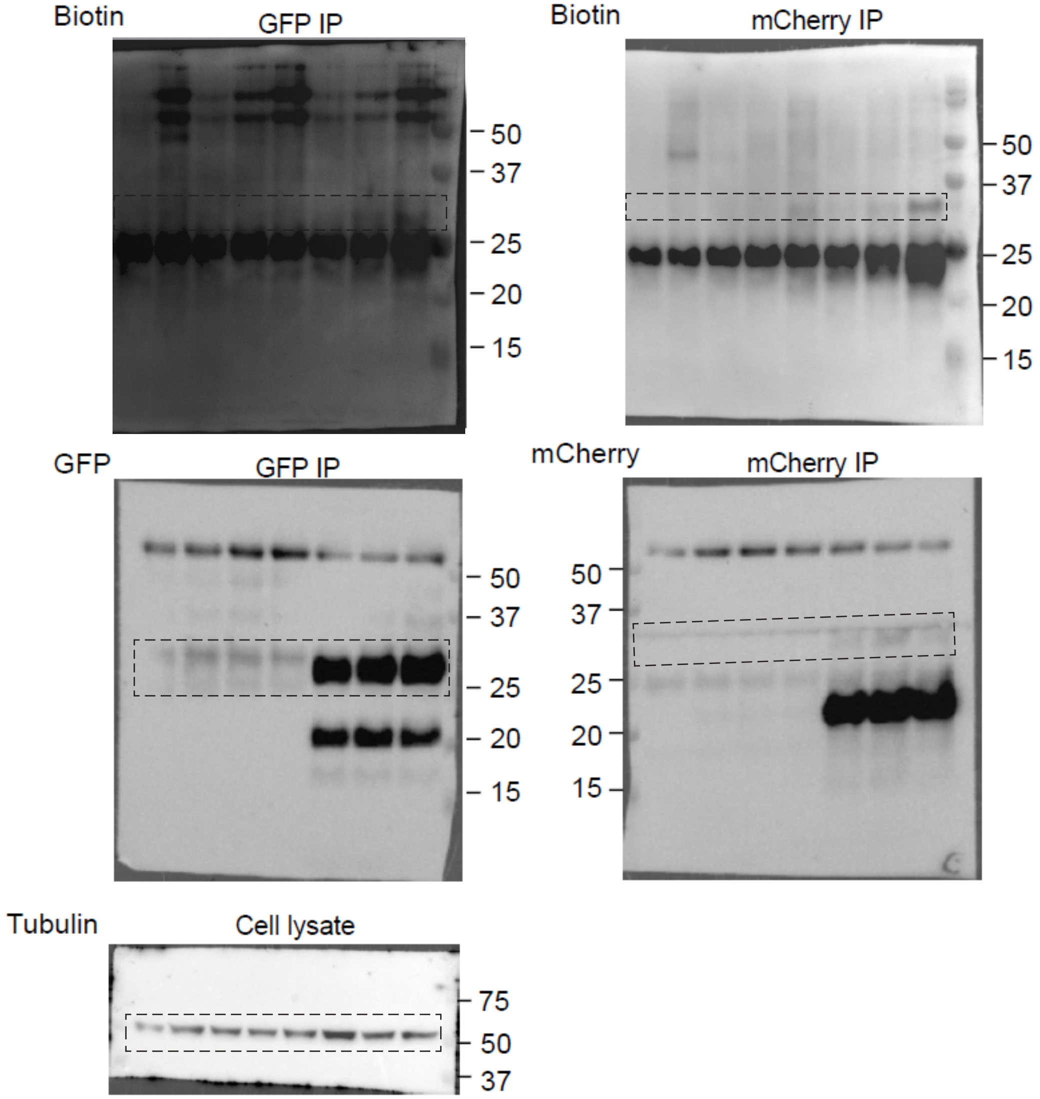


**Supplementary Figure 5, continued. Uncropped blots.**

| Entry Clone | Gene | Mutation | RefSeq | mCherry | GFP | GFP/mCherry | Fold over control |
|---|---|---|---|---|---|---|---|
| R780-E002 | Luciferase | wt | | | | | |
| R780-E003 | MAP2K1 | wt | NM_002755.3 | 5175 | 28972 | 0.178620737 | 0.91381 |
| R780-E004 | KRAS | G12V | NM_004985.3 | 7751 | 40558 | 0.191109029 | 0.9777 |
| R780-E005 | HRAS | G12V | NM_005343.2 | 14831 | 53747 | 0.275940983 | 1.41169 |
| R780-E006 | MAP2K1 | S281D/S222D | NM_002755.3 | 6582 | 33176 | 0.198396431 | 1.01498 |
| R780-E009 | RHEB | Q64L | NM_005614.3 | 5319 | 29544 | 0.180036556 | 0.92105 |
| R780-E010 | CTNNB1 | S22A/S37A/ T41A/S45A | NM_001165902 (Mm) | 6453 | 35132 | 0.183678697 | 0.93968 |
| R780-E011 | GSK3B | K85A | NM_001146156.1 | 5985 | 31659 | 0.189045769 | 0.96714 |
| R780-E012 | CTNNB1 | S33Y | NM_001904.3 | 5178 | 26662 | 0.194208987 | 0.99356 |
| R780-E013 | TGFBR1 | wt | NM_004612.2 | 5102 | 27405 | 0.186170407 | 0.95243 |
| R780-E014 | BCL2 | wt | NM_000633.2 | 4833 | 25068 | 0.192795596 | 0.98633 |
| R780-E015 | BCL2L1 | wt | NM_138578.1 | 4637 | 23489 | 0.197411554 | 1.00994 |
| R780-E016 | CASP8 | C360A | NM_033355.3 | 5313 | 26864 | 0.197773973 | 1.01179 |
| R780-E017 | CASP3 | C163A | NM_032991.2 | 4848 | 25714 | 0.188535428 | 0.96453 |
| R780-E018 | TP53 | R175H | NM_001126114.2 | 4484 | 25023 | 0.17919514 | 0.91675 |
| R780-E019 | AKT1 | E17K | NM_005163.2 | 6795 | 34778 | 0.195382138 | 0.99956 |
| R780-E020 | BRAF | V600E | NM_004333.4 | 13523 | 40449 | 0.334322233 | 1.71036 |
| R780-E021 | CCND1 | P287S | NM_053056.2 | 1093 | 6644 | 0.164509332 | 1.054727 |
| R780-E022 | EGFR | L858R | NM_005228.3 | 820 | 286 | 0.348780488 | 1.074195 |
| R780-E024 | FGFR3 | S249C | NM_000142.4 | 1255 | 6934 | 0.180992212 | 1.160405 |
| R780-E025 | FLT3 | D835Y | NM_004119 | 1710 | 6003 | 0.284857571 | 1.826322 |
| R780-E026 | MAP2K1 | P124L | NM_002755.3 | 1049 | 335 | 0.319351764 | 0.983559 |
| R780-E027 | MTOR | S2215Y | NM_004958.3 | 9149 | 764 | 0.083506394 | 1.032618 |
| R780-E029 | NFE2L2 | E79Q | NM_006164.4 | 1363 | 7302 | 0.186661189 | 1.19675 |
| R780-E030 | NRAS | G12D | NM_002524.4 | 2771 | 16194 | 0.171112758 | 1.097064 |
| R780-E031 | NRAS | Q61K | NM_002524.4 | 10620 | 47027 | 0.225827716 | 1.371798 |
| R780-E032 | PDGFRA | E229K | NM_006206.4 | 817 | 282 | 0.345165239 | 1.063061 |
| R780-E033 | PIK3CA | E545K | NM_006218.2 | 7832 | 675 | 0.086184883 | 1.06574 |
| R780-E034 | PIK3CA | H1047R | NM_006218.2 | 1215 | 7030 | 0.172830725 | 1.108078 |
| R780-E037 | PTEN | R130Q | NM_000314.4 | 11298 | 809 | 0.071605594 | 0.885456 |
| R780-E039 | STK11 | D194N | NM_000455 | 1192 | 6673 | 0.178630301 | 1.145262 |
| R780-E040 | KRAS | G12C | NM_004985.3 | 6067 | 36722 | 0.165214313 | 1.0036 |
| R780-E041 | KRAS | G12D | NM_004985.3 | 2661 | 16727 | 0.159084116 | 1.019944 |
| R780-E042 | KRAS | G13D | NM_004985.3 | 2512 | 6380 | 0.393730408 | 0.961762 |
| R780-E043 | KRAS | Q61L | NM_004985.3 | 960 | 409 | 0.426041667 | 1.312149 |
| R780-E044 | KRAS | Q61R | NM_004985.3 | 2362 | 6329 | 0.373202718 | 1.564193 |
| R780-E045 | CTNNB1 | S37C | NM_001165902.1 | 6889 | 676 | 0.09812745 | 1.213419 |
| R780-E046 | BAP1 | E31A | NM_004656.3 | 2034 | 8222 | 0.247385064 | 1.036857 |

| | | | | | | | |
|---|---|---|---|---|---|---|---|
| R780-E047 | CDH1 | D254Y | NM_004360.4 | 5959 | 38643 | 0.154206454 | 0.93673 |
| R780-E048 | EZH2 | Y646N | NM_004456 | 1278 | 4726 | 0.270418959 | 1.1334 |
| R780-E049 | GATA2 | H460P | NM_032638.4 | 1138 | 5454 | 0.208654199 | 0.87453 |
| R780-E051 | IDH1 | R132H | NM_005896 | 1905 | 5498 | 0.346489633 | 1.45223 |
| R780-E053 | MLH1 | R217C | NM_000249.3 | 1307 | 7381 | 0.177076277 | 1.1353 |
| R780-E054 | MSH2 | Q419K | NM_000251.2 | 5787 | 32458 | 0.178291947 | 1.08304 |
| R780-E055 | MYD88 | L265P | NM_002468.4 | 1749 | 7158 | 0.244341995 | 1.56656 |
| R780-E056 | RET | M918T | NM_020630.4 | 1323 | 8093 | 0.163474608 | 1.04809 |
| R780-E057 | STAG2 | R370W | NM_006603.4 | 1327 | 5547 | 0.239228412 | 1.00267 |
| R780-E058 | U2AF1 | S34F | NM_006758 | 1351 | 7413 | 0.182247403 | 1.16845 |
| R780-E059 | SMAD4 | R361H | NM_005359.5 | 1219 | 4638 | 0.262828806 | 1.10159 |
| R780-E060 | TET2 | S268A | NM_001127208.2 | 9423 | 691 | 0.073331211 | 0.90679 |
| R780-E061 | JAK3 | L527R | NM_000215.3 | 1308 | 7665 | 0.170645793 | 1.09407 |
| R780-E062 | MAP2K4 | R134W | NM_003010.3 | 1074 | 6413 | 0.167472322 | 1.07372 |
| R780-E063 | RUNX1 | D198G | NM_001754.4 | 1209 | 6073 | 0.199077886 | 0.83439 |
| R780-E065 | VHL | N78D | NM_000551.3 | 1689 | 8860 | 0.190632054 | 1.22221 |
| R780-E066 | KLF4 | K409Q | NM_004235.5 | 1780 | 8469 | 0.210178297 | 1.34753 |
| R780-E069 | CYLD | G160R | NM_001042412.1 | 7036 | 729 | 0.103610006 | 1.28121 |
| R780-E070 | SMAD2 | R321Q | NM_005901.5 | 6067 | 34426 | 0.17623308 | 1.07053 |
| R780-E071 | ACVR1B | R484H | NM_004302.4 | 1292 | 7308 | 0.176792556 | 1.13348 |
| R780-E072 | HGF | E199K | NM_000601.5 | 4065 | 641 | 0.157687577 | 1.02042 |
| R780-E073 | BCL2 | R98H | NM_000633.2 | 4350 | 700 | 0.16091954 | 1.04134 |
| R780-E075 | CHEK2 | K373E | NM_007194.3 | 4284 | 671 | 0.156629318 | 1.01358 |
| R780-E076 | KEAP1 | R470C | NM_203500.1 | 4827 | 700 | 0.145017609 | 0.93844 |
| R780-E077 | FOXA1 | I176M | NM_004496.3 | 3701 | 563 | 0.152121048 | 0.9844 |
| R780-E079 | EIF4A2 | G336W | NM_001967.3 | 4525 | 688 | 0.152044199 | 0.98391 |
| R780-E080 | ELF3 | S133F | NM_004433.4 | 5328 | 33130 | 0.160821008 | 0.97691 |
| R780-E081 | HIST1H1C | K109N | NM_005319.3 | 4192 | 665 | 0.158635496 | 1.02656 |
| R780-E082 | CDKN1B | D63E | NM_004064.4 | 2160 | 5027 | 0.429679729 | 1.04957 |
| R780-E083 | CBFB | R151H | NM_001755.2 | 5540 | 34261 | 0.161699892 | 0.98225 |
| R780-E084 | CDKN1A | W49L | NM_000389.4 | 4200 | 670 | 0.15952381 | 1.03231 |
| R780-E085 | H3F3B | G35R | NM_005324.4 | 4139 | 617 | 0.149069824 | 0.96466 |
| R780-E086 | GNAS | R201H | NM_000516.5 | 12413 | 76364 | 0.162550416 | 0.98742 |
| R780-E087 | B4GALT3 | H194Y | NM_003779.3 | 5821 | 37704 | 0.154386802 | 0.93783 |
| R780-E088 | SPOP | F133L | NM_003563.3 | 5344 | 32796 | 0.162946701 | 0.98982 |
| R780-E089 | TSC1 | R786Q | NM_000368 | 21888 | 1617 | 0.073876096 | 0.91353 |

**Supplementary Table 1.** Summary of the Cancer Toolkit (CTK) lentiviral library screen.
